# A Labeling Strategy for Living Specimens in Long-Term/Super-Resolution Fluorescence Imaging

**DOI:** 10.3389/fchem.2020.601436

**Published:** 2021-01-15

**Authors:** Yubing Han, Zhimin Zhang, Wenjie Liu, Yuanfa Yao, Yingke Xu, Xu Liu, Cuifang Kuang, Xiang Hao

**Affiliations:** ^1^State Key Laboratory of Modern Optical Instrumentation, College of Optical Science and Engineering, Zhejiang University, Hangzhou, China; ^2^Department of Biomedical Engineering, Key Laboratory of Biomedical Engineering of Ministry of Education, Zhejiang Provincial Key Laboratory of Cardio-Cerebral Vascular Detection Technology and Medicinal Effectiveness Appraisal, Zhejiang University, Hangzhou, China; ^3^Ningbo Research Institute, Zhejiang University, Ningbo, China; ^4^Collaborative Innovation Center of Extreme Optics, Shanxi University, Taiyuan, China

**Keywords:** super-resolution microscopy, organic fluorescent dye, long-term imaging, living specimens, subcellular structures

## Abstract

Despite the urgent need to image living specimens for cutting-edge biological research, most existing fluorescent labeling methods suffer from either poor optical properties or complicated operations required to realize cell-permeability and specificity. In this study, we introduce a method to overcome these limits—taking advantage of the intrinsic affinity of bright and photostable fluorophores, no matter if they are supposed to be live-cell incompatible or not. Incubated with living cells and tissues in particular conditions (concentration and temperature), some Atto and BODIPY dyes show live-cell labeling capability for specific organelles without physical cell-penetration or chemical modifications. Notably, by using Atto 647N as a live-cell mitochondrial marker, we obtain 2.5-time enhancement of brightness and photostability compared with the most commonly used SiR dye in long-term imaging. Our strategy has expanded the scientist's toolbox for understanding the dynamics and interactions of subcellular structures in living specimens.

## Introduction

Biologists rely on an array of fluorescent microscopy to observe morphologies and dynamics in living cells, which is crucial in interpreting vital physiological and pathological activities. However, although dramatic improvements have been implemented since the seminal discovery of fluorophores and their applications in microscopy (Miyawaki et al., 2003; Specht et al., 2017), it is still quite challenging to achieve live-cell specific staining simultaneously with high brightness and photostability.

In fact, most live-cell labeling methods, including fluorescent proteins (Mishin et al., 2015), chemical tag techniques using cell-permeable fluorescent dyes [e.g., SNAP-Cell 647-SiR (Lukinavičius et al., 2015)], and live-cell organic fluorescent probes [e.g., MitoTracker dyes (Chazotte, 2011)], suffer from relatively low brightness and photostability (Fernandez-Suarez and Ting, 2008; Han et al., 2017). Low optical properties of the fluorescent probes may lead to low signal-to-noise ratios (SNR) and fast photo-bleaching, while low SNR reduces the quality of the microscope images or may even introduce artifacts. Worse still, is that the photobleaching is undesirable in long-term experiments. A dosage increase can partially remedy this problem, but it may also lead to non-specific labeling (Han et al., 2017; Shen et al., 2018). Another option is to increase the illumination laser power, but it, in turn, further accelerates the photobleaching and introduces stronger phototoxicity. This issue stands out especially in imaging methods for more information in multiple dimensions (e.g., high spatiotemporal resolution, long acquisition times, and large volume imaging) (Jaiswal et al., 2003; Fernandez-Suarez and Ting, 2008; Ji et al., 2016).

In the past few decades, various types of commercially available dyes, with exceptionally excellent brightness and photostability, have been developed and are widely used in cells and organisms (Chazotte, 2011; Sigal et al., 2018). Although several dyes have been proven to be efficient in live-cell super-resolution imaging (Lukinavicius et al., 2014; Yang et al., 2020), most of these fluorophores were supposed to be “live-cell incompatible” (Mao et al., 2020). Their intracellular cytosolic delivery and labeling specifically, rely heavily on specific physical or chemical methods, such as microinjection, encapsulating vesicles, or chemical modifications using cell-penetrating peptides (Erazo-Oliveras et al., 2014; Hennig et al., 2015; Han et al., 2017).

Following our previous work, which demonstrated the live-cell mitochondrial labeling capacity of Atto 647N (Han et al., 2017), here we prove that Atto 647N is not an individual case and that there is a general pattern behind it. We present a strategy to label living cells and tissues using commercially available dyes that were supposed to be “incompatible” for live-cell labeling (von Provazek, 1914; Bosch et al., 2014). The transition from “incompatible” to “compatible” is based on discovering and utilizing the intrinsic affinity of the dyes to subcellular structures in living cells when working at micromolar-level concentrations. Specifically, by incubating with living specimens at 1.5–15 μM for 30 min at 37°C or 20°C, the “live-cell incompatible” dyes can also be used for live-cell labeling, without needing complicated chemical modifications or physical operations during the entire process. Using our labeling strategy, these commercially available dyes, especially Atto 647N NHS ester, shows great potential as a live-cell mitochondrial marker in long-term, three-dimensional (3D), and super-resolution imaging.

## Materials and Methods

### Primary Cultural Astrocytes

Primary cultural astrocytes were obtained from Sprague-Dawley (SD) rat brains (1 day old). The rats were bought from the Zhejiang Research Center of Laboratory Animals, China, sterilized with 75% ethanol, and sacrificed by decapitation. The following steps were all done on ice. The scalps and skulls were incised, and the brains were taken out and placed into pre-cooling Phosphate buffered saline (PBS; Thermo Fisher Scientific, Inc.). The cortex was then isolated, cut into pieces using knives, and incubated with pre-warmed Trypsin-EDTA (Thermo Fisher Scientific, Inc.) at 37°C for 20 min. The mixture was centrifuged for 5 min at 1,000 rpm, and the supernatant was removed. The cells were then dissociated by adding 5 mL of pre-warmed growth medium (DMEM, high glucose + 10% fetal bovine serum (FBS); Thermo Fisher Scientific, Inc.), followed by vigorous pipetting. Finally, the cell suspension was incubated in a T25 flask (Thermo Fisher Scientific, Inc.) at 37°C in a humidified 5% CO_2_ environment. The medium was changed every 2 days, and after 7 ~ 8 days, the culture flask was shaken manually for 30 min to remove the overlaying microglia exposed on the astrocyte layer. The supernatant containing microglia was discarded, and 5 mL of culture medium was added into the flask. This step was repeated once to remove oligodendrocyte precursor cells.

### Cell Culture

HeLa (human adenocarcinoma cell line), HEK293 (human embryonic kidney epithelial cell line), NIH/3T3 (mouse embryonic fibroblast cell line), and U2OS (human osteosarcoma cell line) cells were purchased from the American Type Culture Collection. HeLa, HEK293, and NIH/3T3 cells were cultured in the DMEM medium (Thermo Fisher Scientific, Inc.). U2OS cells were cultured in McCoy's 5A medium (Thermo Fisher Scientific, Inc.). All media were supplemented with 10% (v/v) FBS, and the cultures were maintained at 37°C in a humidified 5% CO_2_ environment.

### Transfection

Cells were grown overnight in 24-well plates at 37°C in a 5% CO_2_ atmosphere. After reaching over 80% confluence, the plasmids mRuby-Clathrin (Addgene #55852), pEGFP-Sec23A (Addgene #66609), ZsGreen-Rab5 (custom synthesized by Genomeditech (Shanghai, China) Co., Ltd.), EGFP-Rab7A (Addgene #28047), GFP-LAMP1 (Addgene #16290), EMTB-3XGFP (Addgene #26741), pSNAPf-Cox8A (Addgene #101129), or pSNAPf-TOMM20 (custom synthesized by Genomeditech (Shanghai, China) Co., Ltd.) was transfected into the cells using Lipofectamine 3000 (Thermo Fisher Scientific, Inc.) according to the manufacturer's instructions. After 24 h, the transfected cells were digested with trypsin-EDTA and seeded into Nunc Glass Bottom Dishes (Φ 12 mm, Thermo Fisher Scientific, Inc.) at a density of 1.5 ~ 2.0 × 10^4^ per well in growth medium (150 μL). The cells were grown for an additional 12 ~ 24 h before incubation with the indicated probes.

### Live-Cell Labeling With Commercially Available Probes

Before staining, the indicated cells were seeded in Nunc Glass Bottom Dishes at a density of 1.5 ~ 2.0 × 10^4^ per well in growth medium (150 μL). After overnight incubation, the cells were washed three times with PBS. Working solutions of the indicated probes at different concentrations were prepared with phenol red-free DMEM (Thermo Fisher Scientific, Inc.). The cells were then incubated with MitoTracker Deep Red FM (200–500 nM, 100 μL; Thermo Fisher Scientific, Inc.), MitoTracker Green FM (1,000 nM, 100 μL; Thermo Fisher Scientific, Inc.), SiR-actin (Cytoskeleton, Inc.), and DiO (5 μM, 100 μL; Beyotime Biotechnology) in a 5% CO_2_ atmosphere at 37°C for 30 min. For SNAP-Cell 647-SiR (New England Biolabs, Inc.) dye labeling, transfection of pSNAPf-Cox8A or pSNAPf-TOMM20 was performed before incubation of SNAP-Cell 647-SiR (3 μM) at 37°C for 30 min. After incubation, the supernatant was discarded, and a solution of Trypan blue (TB, 100 μL, 1 mg mL^−1^; Sigma-Aldrich Co., LLC) in PBS was added to exclude the dead cells and quench the extracellular fluorescence from the probes bound to either the cell membrane or the dish surface (Manceur et al., 2007; Cottet-Rousselle et al., 2011; Han et al., 2017). After 1 min, TB was removed, and the cells were washed twice gently with PBS. Cells were immersed in Live Cell Imaging Solution (Thermo Fisher Scientific, Inc.) before imaging. ProLong Live Antifade Reagent (Thermo Fisher Scientific, Inc.) was added to this solution according to the manufacturer's instructions when required.

### Live-Cell Labeling With Fluorescent Dyes

Before staining, the indicated cells were seeded in Nunc Glass Bottom Dishes at a density of 1.5 ~ 2.0 × 10^4^ per well in growth medium (150 μL). After overnight incubation, the cells were washed three times with PBS. Aliquots of Atto 495, Atto 565, Atto 590, Atto 647N (Sigma-Aldrich Co., LLC), BODIPY 650/665, Alexa Fluor 647 (Thermo Fisher Scientific, Inc.), Cy3B, and Cy5 (GE Healthcare Co., Ltd) NHS esters were dissolved in Dimethyl sulfoxide (DMSO; Sigma-Aldrich Co., LLC) to make 3-mM stock solutions at −20°C. Stock solutions were diluted with phenol red-free DMEM to work solutions at different concentrations before use. The cells were incubated with these dyes either at 37°C in a 5% CO_2_ atmosphere or at 20°C for 30 min. The post-incubation treatment was the same as that of the commercially available probes above.

The organic probes Dye-Lyso, Dye-Tubulin, and Dye-Actin were constructed in two parts. One part contains the recognition unit (epoxysuccinyl scaffold (Han et al., 2017), docetaxel (Lukinavicius et al., 2018), and Lifeact (Han et al., 2019), the cell-penetrating peptide (rR)_3_R_2_, and a short peptide GKGKGK (in which lysine offers free active amino groups available to conjugate with commercially available dyes via an NHS group). The other part is a commercially available fluorescent dye containing an NHS group. Covalent bonds link these two parts to form an entire probe. The first part was later purified by preparative high-performance liquid chromatography (HPLC) to >95% after being prepared by solid-phase peptide synthesis. Its mass was confirmed by electrospray ionization mass spectrometry (EI-MS). Before being conjugated to the dyes, the peptide part was dissolved in bicarbonate buffer (0.1 M, pH 8.3) at a 1 mM concentration and stored at 4°C. For the conjugation, an aliquot of each dye was dissolved with 10–20 μL of DMSO added to 14 μL of the peptide part solution and mixed thoroughly. The mixture was allowed to react at room temperature overnight with constant shaking. The mixture was then purified using Pierce C18 Spin columns (Thermo Fisher Scientific, Inc.) according to the manufacturer's instructions. After purification, the supernatant was evaporated to dryness in a vacuum centrifuge, and the residue was dissolved in 200 μL of Phosphate Buffered Saline (PBS, pH 7.4; Thermo Fisher Scientific, Inc.) to generate a stock solution of the indicated probes.

Filipin (Meilunbio) and Cytochalasin D (Aladdin) were dissolved in DMSO to make 1-mM stock solutions, which were diluted with phenol red-free DMEM to working solutions at different concentrations before use. The cells were incubated with the drug solutions for 30 min at 37°C in a 5% CO_2_ atmosphere before Atto 565 labeling.

### Labeling Living Tissue Slices With Fluorescent Probes

All animal procedures were conducted in compliance with the guidelines for animal care and use of Zhejiang University and conformed to the Guide for the Care and Use of Laboratory Animals published by the National Academy Press (Washington, DC, 1996). The 12-week-old Institute of Cancer Research (ICR) mice were obtained from Zhejiang Academy of Medical Sciences [License Number: SCXK (Zhe) 2014001] and housed in cages under a standard condition of temperature (23 ± 2°C), relative humidity (55 ± 5%), and a light 12/12 h light/dark cycle. The mice had free access to food and water. Before the experiment, the mice were sacrificed by euthanasia. The brains were separated and washed with cold PBS buffer. The brains were then cut into slices from different orientations and incubated with Atto 647N (15 μM) for 30 min at 37°C. After incubation, the supernatant was discarded, and the tissue slices were washed twice gently with PBS. The tissue slices were then put on the Nunc Glass Bottom Dishes with the wound surface toward the glass bottom.

### Cell Viability

The Atto dyes' cytotoxicity on different cell lines was tested using the 3-(4,5-dimethylthiazol-2-yl)-5-(3-carboxymethoxyphenyl)-2-(4-sulfophenyl)-2H-tetrazolium (MTS) assay (Cory et al., 1991). Cells (3–4 × 10^3^ cells per well) were seeded into a 96-well plate and cultured in the growth medium for 24 h. The cells were incubated with the dyes (1.5 μM or 15 μM) in a 5% CO_2_ atmosphere at 20°C or 37°C for 30 min, washed twice gently with PBS, and incubated in the growth medium for 3 h at 37°C. The cells were then immersed in 100 μL of growth medium and 20 μL of CellTiter 96 AQueousOne Solution Reagent (Promega Co.) for another 1 h at 37°C, and the absorbance was recorded at 492 nm using a TECAN GENios Plus ELISA reader (Tecan, Inc.). The cell viabilities were expressed as the percentage of the A492 of the dye-treated cells to the untreated controls. The A492 of the dyes themselves, tested in cells without MTS treats, were subtracted in the treated cells. All the measurements were performed in triplicate.

### pH Sensitivity of Atto 565

Solutions of Atto 565 at different pH were diluted from the stock solution (see Section Live-cell labeling with fluorescent dyes) with citric acid or sodium hydroxide solutions at certain concentrations (Sigma-Aldrich Co., LLC). The fluorescence intensity was recorded (λ_ex_ = 563 nm) using a SpectraMax M2e (Molecular Devices, LLC.).

### Confocal Laser Scanning Microscopy

The confocal images were obtained using a C2 confocal laser scanning microscope (Nikon, Inc.) equipped with a 100×/1.49 numerical aperture (NA) oil immersion objective lens and were analyzed with NIS-elements (Nikon, Inc.) and ImageJ software (National Institutes of Health).

### STED Microscopy

The STED images were obtained using a STEDYCON microscope (Abberior, GmbH.) equipped with a 100×/1.49 NA oil immersion objective lens. A 40-MHz pulsed laser (775 nm) was used for depletion. The depletion laser intensity on the back-pupil plane was measured as about 80 mW. The depletion time was set as 1.2 ns, and the time gating was set as 1–7 ns. The resolution was calculated with the system's built-in software as 37 nm, using 40-nm red fluorescent beads (Abberior, GmbH.). Pinhole: 64 μm. For **Figure 4B**, the pixel size is 50 nm, and the pixel dwell time is 4 μs; for **Figures 4C,E**, the pixel size is 20 nm, and the pixel dwell time is 10 μs (STED) and 10 μs (Confocal).

## Results

### Evaluation of Atto Dyes for Labeling Living Cells

Several types of fluorescent dyes were characterized according to their structure series.

Many cell-permeable cationic dyes (e.g., Rhodamine and Carbocyanine derivates) have been developed as mitochondrial probes, as they tend to accumulate in the mitochondrial matrix driven by the potential gradient in mitochondria at about 100-nM concentrations (Chazotte, 2011; Cottet-Rousselle et al., 2011; Xu et al., 2016). Endoplasmic reticulum (ER) staining can also be realized by raising the dosages of these dyes (Han et al., 2017). However, for the “live-cell compatible” dyes, concentrations at the 100-nM level are not enough.

**Figure 1:**
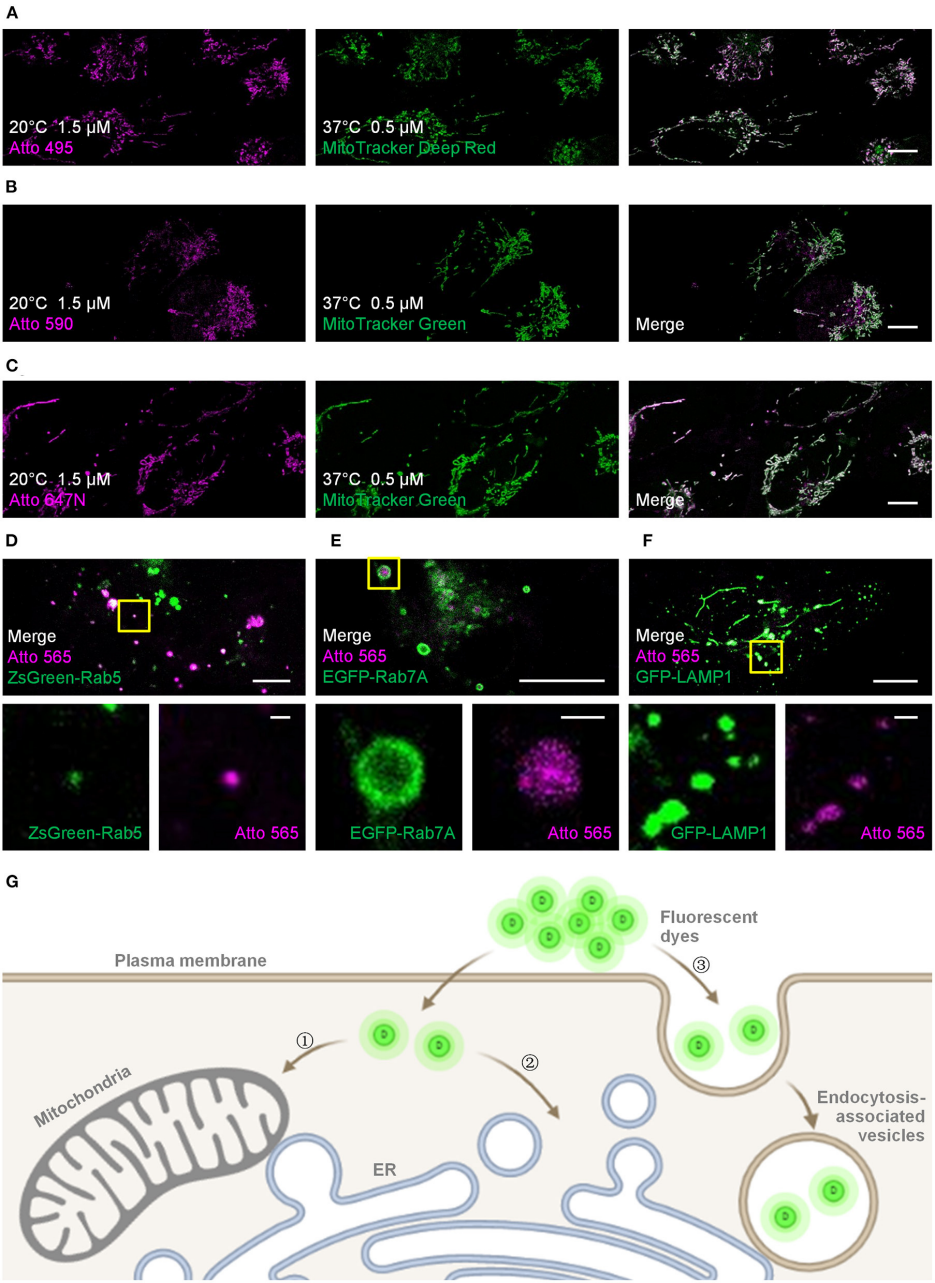
Live-cell labeling of the Atto dyes. Colocalization studies employing different fluorescent probes and proteins as the standard mitochondrial markers. **(A)** Living U2OS cells were incubated with Atto 495 (magenta, 1.5 μM) for 30 min at 20°C and then with MitoTracker Deep Red (green, 0.5 μM) for 30 min at 37°C before imaging. Living U2OS cells were incubated with **(B)** Atto 590 or **(C)** Atto 647N (magenta, 1.5 μM) for 30 min at 20°C and then with MitoTracker Green (green, 0.5 μM) for 30 min at 37°C before imaging. Pearson correlation coefficient: 0.70, 0.57, and 0.82 for Atto 495, Atto 590, and Atto 647N, respectively. Living U2OS cells transiently transfected by **(D)** ZsGreen-Rab5 (green; early endosomes), **(E)** EGFP-Rab7A (green; late endosomes), and **(F)** GFP-LAMP1 (green; lysosomes) were stained with Atto 565 (magenta, 6 μM) for 30 min at 37°C and imaged by confocal microscope. Top panels: merged channels; bottom panels: enlarged single-channel images from the yellow boxed regions in the top panels, revealing colocalization of Atto 565 and specific endocytic vesicles. **(G)** The dyes enter living cells through , concentration-dependent free diffusion (e.g., Atto 647N), or endocytosis (e.g., Atto 565). Scale bars, (**A–C**, upper panels of **D–F**) 10 μm, and (lower panels of **D–F**) 1 μm.

Four Rhodamine derivatives, i.e., Atto 495, Atto 565, Atto 590, and Atto 647N N-Hydroxysuccinimide (NHS) esters, were investigated due to their relatively good optical properties in the corresponding spectral bands (Figure 1; Supplementary Figures 1–6). When incubated at 37°C, which is the optimal temperature for cell cultures, both mitochondria-like and bright dot signals inside the cells were observed (Supplementary Figure 1B); whereas when the incubation temperature was set to 20°C (room temperature), the dot signals were dramatically reduced (Supplementary Figure 1C).

For the mitochondria-like signals, colocalization experiments using MitoTracker probes were performed to verify the mitochondrial staining, and the Pearson correlation coefficients for Atto 495, Atto 590, and Atto 647N were 0.70, 0.57, and 0.82, respectively (Figures 1A–C; Supplementary Figure 2). The results indicate the mitochondrial labeling of Atto 495 and Atto647N.

Furthermore, the dot signals of Atto 565 were partially colocalized with endocytic-associated vesicles (i.e., early endosomes, late endosomes, and lysosomes; Figures 1D,F and Supplementary Figures 3–5), while no macroscopic colocalization was found with endocytic-unassociated vesicular structures (Supplementary Figure 6). After the cells were treated with Filipin and Cytochalasin D, which are known as endocytosis inhibitors (Rodal et al., 1999; Fujimoto et al., 2000; Dutta and Donaldson, 2012), the fluorescence intensity of Atto 565 labeling decreased to 36 ~ 68% (Supplementary Figure 7). These results suggest the dependence of the dot signals on endocytosis to some extent (Figure 1G). The pH sensitivity of Atto 565 was also investigated since most of its signals were discovered in vesicles with an acidic environment (Yamashiro et al., 1983). The results showed that the fluorescence intensity of Atto 565 decreases as the pH values decline, suggesting that its brightness is limited when caught in endocytic vesicles (Supplementary Figure 8).

### Staining Mechanism of Atto 647N and 565 NHS Ester

**Figure 2:**
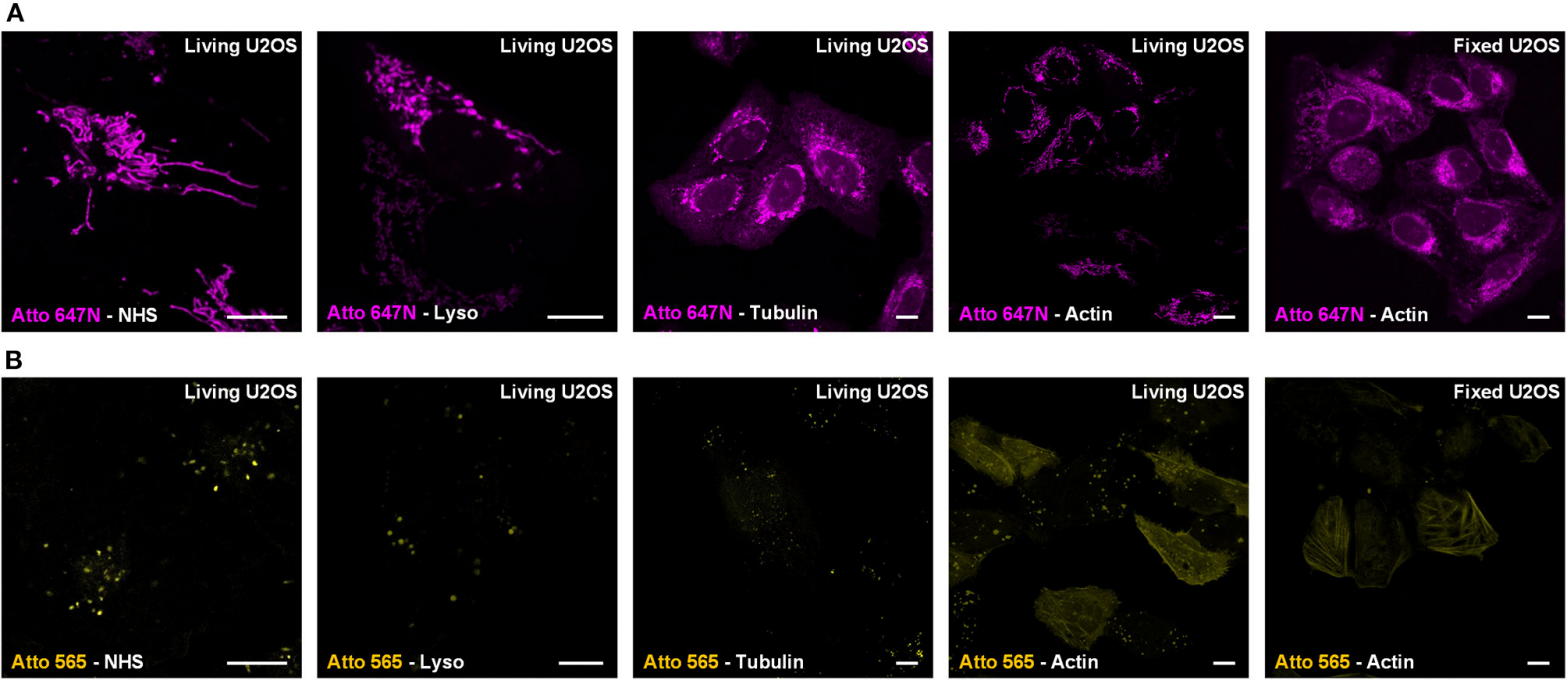
Live-cell labeling mechanism of Atto 647N and Atto 565. The dyes were conjugated with either NHS ester (Dye-NHS) or different recognition units targeting lysosomes (Dye-Lyso), microtubules (Dye-Tubulin), and actin filaments (Dye-Actin). Confocal images show the intracellular distribution of probes containing **(A)** Atto 647N and **(B)** Atto 565 in living or fixed U2OS cells.

For Atto 647N NHS ester, which contains two parts (i.e., the cationic dye Atto 647N and the NHS moiety), we further investigated its mitochondrial labeling mechanism. Various recognition units (Han et al., 2017) were conjugated to Atto 647N to verify the dye's affinity to mitochondria. Probes were named as Atto 647N -Lyso, Atto 647N -Tubulin, and Atto 647N -Actin according to the recognition units they contain (Figure 2A). Atto 647N NHS ester was taken as a control group. The results showed that the conjugated units did not work, and all the probes labeled mitochondria or ER. Similar tests were performed for Atto 565, too. The results showed that only Atto 565-Actin partially marked actin filaments, and the other Atto 565-probes only labeled vesicle structures (Figure 2B). These results suggest that the affinity competition occurs between the dyes and their conjugated groups, and there is a strong tendency for mitochondria in Atto 647N itself, no matter what ligands were bound to it.

Fluorescent cationic dyes that tend to accumulate in mitochondria can be divided into two categories. Without any other active groups (e.g., Rhodamine 123), dyes may fail to stain mitochondria after fixation due to the loss of membrane potential, while with active groups (e.g., MitoTracker probes), dyes can form covalent bonds with mitochondrial membrane proteins (Poot et al., 1996). In our previous tests (Han et al., 2017), Atto 647N NHS ester kept its labeling pattern after fixation, indicating that the NHS ester contributes to the labeling by covalently binding to mitochondrial membrane proteins. The colocalization study employing MitoTracker Green by super-resolution imaging, which showed a high fluorescent signal in both inner and outer mitochondrial membranes, further proved this (Han et al., 2017). A recent labeling method, named FLARE also utilizes Atto 647N NHS ester as a mitochondrial marker in fixed cells by the reaction with amines (Mao et al., 2020).

The above results indicate that both the cationic charge of Atto 647N and the covalent bonds from the NHS ester contribute to the mitochondrial affinity of Atto 647N NHS ester.

### Evaluation of the Dyes From Other Series for Live-Cell Labeling

Then, as Carbocyanine dyes, anionic Alexa Fluor (AF) 647, zwitterionic Cy3B, and cationic Cy5 were chosen as representative research objects (Supplementary Figures 9A–C). No mitochondrial staining was found after the incubation at either 37°C or 20°C. For AF 647 and Cy3B, the introduction of sulfonic acid groups improves fluorescence and solubility in water but adds negative charges, preventing their affinity for mitochondria (Wories et al., 1985). For Cy5, the high hydrophobicity leads to a certain degree of affinity for the plasma membrane (Pearson correlation coefficient: 0.52; Supplementary Figures 9B, 10A), preventing its permeation into living cells (Simons et al., 2009).

Unexpectedly, zwitterionic BODIPY 650/665 showed an affinity for mitochondria at a low concentration (300 nM, Pearson correlation coefficient: 0.52; Supplementary Figures 9D, 10B) while labeled ER at a relatively high concentration (15 μM, Pearson correlation coefficient: 0.82; Supplementary Figures 9D, 10C) was shown, suggesting its potential as a live-cell ER marker. Our results also indicate the mitochondrial affinity of BODIPY 650/665, breaking through the earlier conclusion (Xu et al., 2016) that this purpose can only be achieved using cationic dyes.

In view of the above observations, we hypothesize that without additional vector reagents and physical penetration, a group of fluorescent dyes, no matter if they were initially considered live-cell compatible or not, can mark subcellular structures in living cells using our labeling strategy (Supplementary Table 1).

### Labeling in Various Types of Living Cells

**Figure 3:**
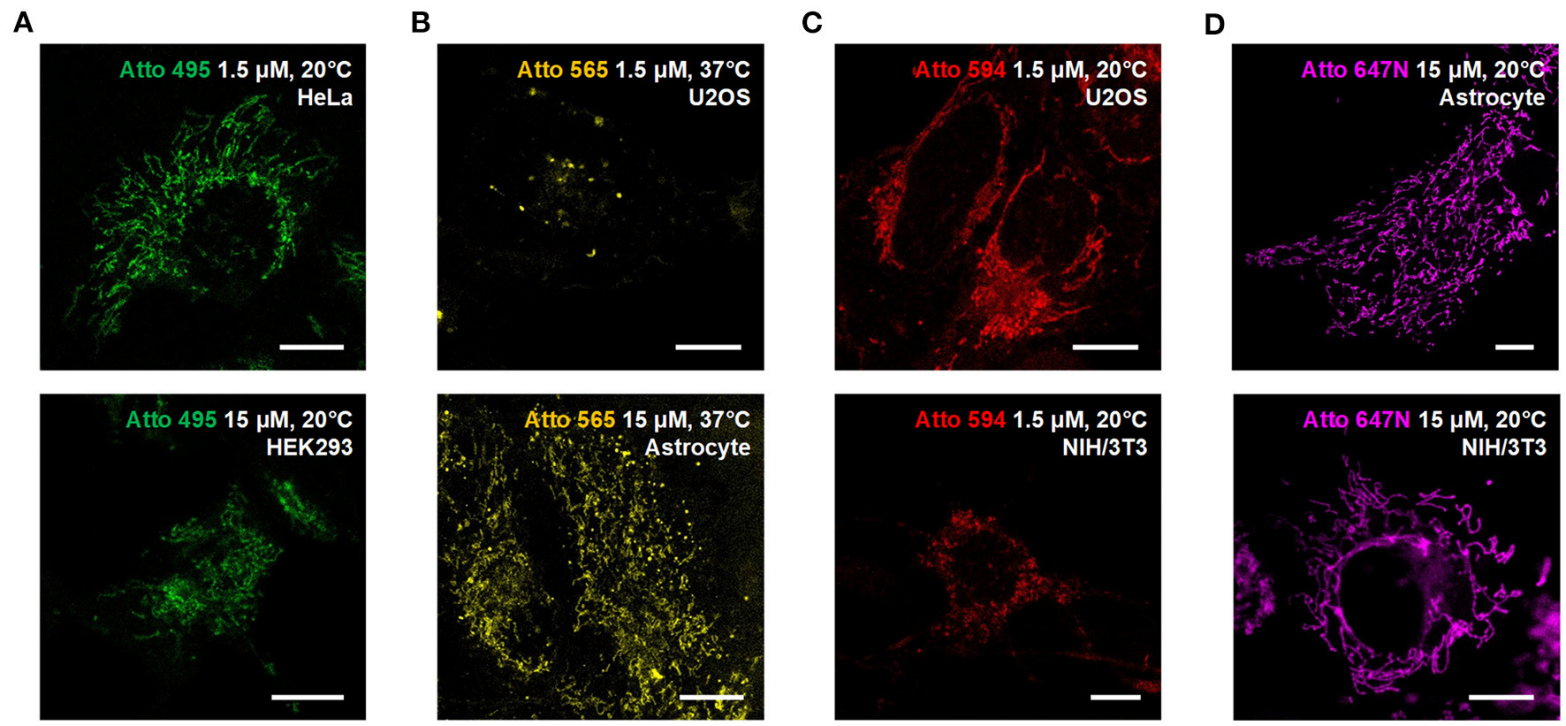
Compatibility of the Atto dyes to various types of living cells. Confocal images of living U2OS, Astrocyte, H3K293, HeLa, and NIH/3T3 cells incubated with **(A)** Atto 495, **(B)** Atto 565, **(C)** Atto 590, and **(D)** Atto 647N at either 37°C or 20°C. Scale bars, 10 μm.

We also used the living HeLa (human adenocarcinoma cell line), HEK293 (human embryonic kidney epithelial cell line), NIH/3T3 (mouse embryonic fibroblast cell line) cells, and primary cultural astrocytes from 1-day-old Sprague-Dawley (SD) rats (Figure 3; Supplementary Figure 11) to demonstrate the universality of our strategy. Notably, while the transfection in primary cultural astrocytes is challenging to achieve, the incubation with fluorescent probes is quite efficient and straightforward.

Compared with the above-mentioned fluorescent probes containing fluorescent dyes, recognition units, and cell-permeable peptides, which also work at the micromolar level (Han et al., 2017), the cytotoxicity of our method here mainly comes from the dyes themselves and the organic solvent in the incubation buffer (e.g., DMSO). To further confirm that our approach is compatible with the living specimens, the cytotoxicity of this method was then investigated by a 3-(4,5-dimethylthiazol-2-yl)-5-(3-carboxymethoxyphenyl)-2-(4-sulfophenyl)-2H-tetrazolium (MTS) assay (Cory et al., 1991). The cell viabilities were expressed as the percentage of the dye-treated cells' absorbance to the untreated controls. All the measurements were performed in triplicate. The results showed that after being treated with the indicated dyes, the cell viabilities usually exceeded 80% when incubated at 37°C and between 60 and 90% when incubated at 20°C, except in isolated cases (Supplementary Figure 12).

### Photostability of Atto 647N

**Figure 4:**
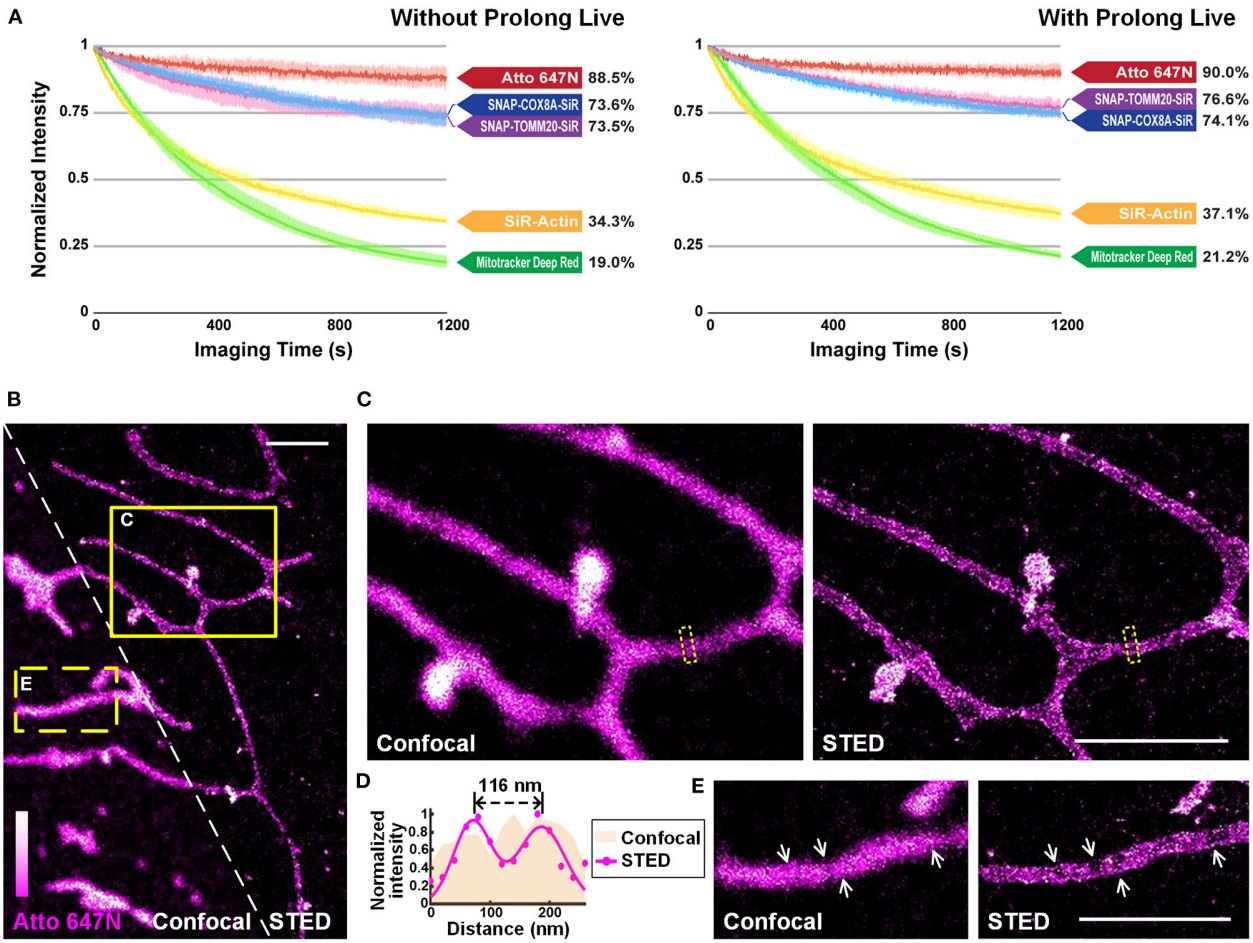
Atto 647N for live-cell imaging applications with high demand for optical properties. **(A)** Curves of intensity decay of the indicated dyes without (left) or with (right) the addition of Prolong Live under the same confocal imaging condition. Consecutive frames spaced at 1-s intervals were recorded. Error bars represent the standard deviations of triplicate experiments. **(B)** Living U2OS cells were incubated with Atto 647N for 30 min at 20°C. Enlarged confocal and STED images from the **(C)** solid or **(E)** dashed yellow boxes shown in **(B)**. **(D)** Intensity profiles at the positions within the dashed boxes in **(C)** (pink area for the confocal image, and magenta dots and Gaussian fitting curve for STED image). Scale bars, 2 μm.

We quantitatively compared the optical properties of the frequently-used red-absorbing fluorophores. For photostability evaluation, fluorescence intensity curves were extracted from 20-min confocal imaging of U2OS cells (Figure 4A; Supplementary Figure 13), where all the imaging settings (e.g., laser power, size of field-of-view, imaging speed) were retained to control variables (Supplementary Table 2). The power density on the sample was adjusted to ~1.25 kW cm^−2^ to ensure that all the probes were bright enough in the first frame. For Atto 647N, whose brightness is 2.50 times that of the SiR dye (Mishin et al., 2015) according to the manufacturers (Supplementary Table 3), the laser power used can be reduced by at least an order of a magnitude. The results showed that: (i) Atto 647N kept more than 88% of its intensity after 20-min of imaging (Supplementary Figure 13A); (ii) incubation of MitoTracker Deep Red at 200 nM (recommended concentration according to the manufacturer's instructions) already exhibited intense non-specific labeling of ER, while the signal intensity of both mitochondria and ER declined rapidly with only 19% left in the end (Supplementary Figure 13B); (iii) SNAP-Cell 647-SiR kept more than 70% of the intensity in the last frame. However, the cells stained with SNAP-Cox8A-SiR exhibited strong background in the cytosols (Supplementary Figures 13C,D); (iv) SiR-Actin were bleached very quickly, indicating the insufficient anti-bleaching property of SiR dye itself (Supplementary Figure 13E). The excess over-expression level of the SNAP- protein, which is relatively challenging to control in normal cancer cells and to realize in primary cultural cells, may result in the low image quality in the SNAP-tag technique (Supplementary Figures 13C,D). However, fluorescent probes are easy to control by simply changing the incubation concentration. The results suggest that Atto 647N exhibited the best photostability (4.66 times the value of MitoTracker Deep Red, and 2.58 times that of SiR dye itself; Figure 3A; Supplementary Figure 13A). By adding antioxidant reagent Prolong Live (Westbrook et al., 2018), all the bleaching speeds of these dyes decelerated to some extent. However, the difference between the dyes remained unchanged (Figure 4A; Supplementary Figure 13).

The performance of Atto 647N was further investigated using stimulated emission depletion (STED) microscopy (Figures 4B–E). The distance of mitochondrial outer membranes was resolved as 116 nm in STED mode, while the confocal image showed blurred structures (Figures 4C,D). Besides, structures of folded inner membranes, cristae, which were arranged in groups, and the voids between them were visible in the STED image (white arrows in Figure 4E) (Han et al., 2017; Huang et al., 2018; Stephan et al., 2019). Therefore, these results indicate that by applying our method in live-cell mitochondrial staining, especially in long-term or super-resolution imaging, Atto 647N can substitute SiR dye (Stephan et al., 2019) by offering better optical properties.

### Dual-Color Applications

**Figure 5:**
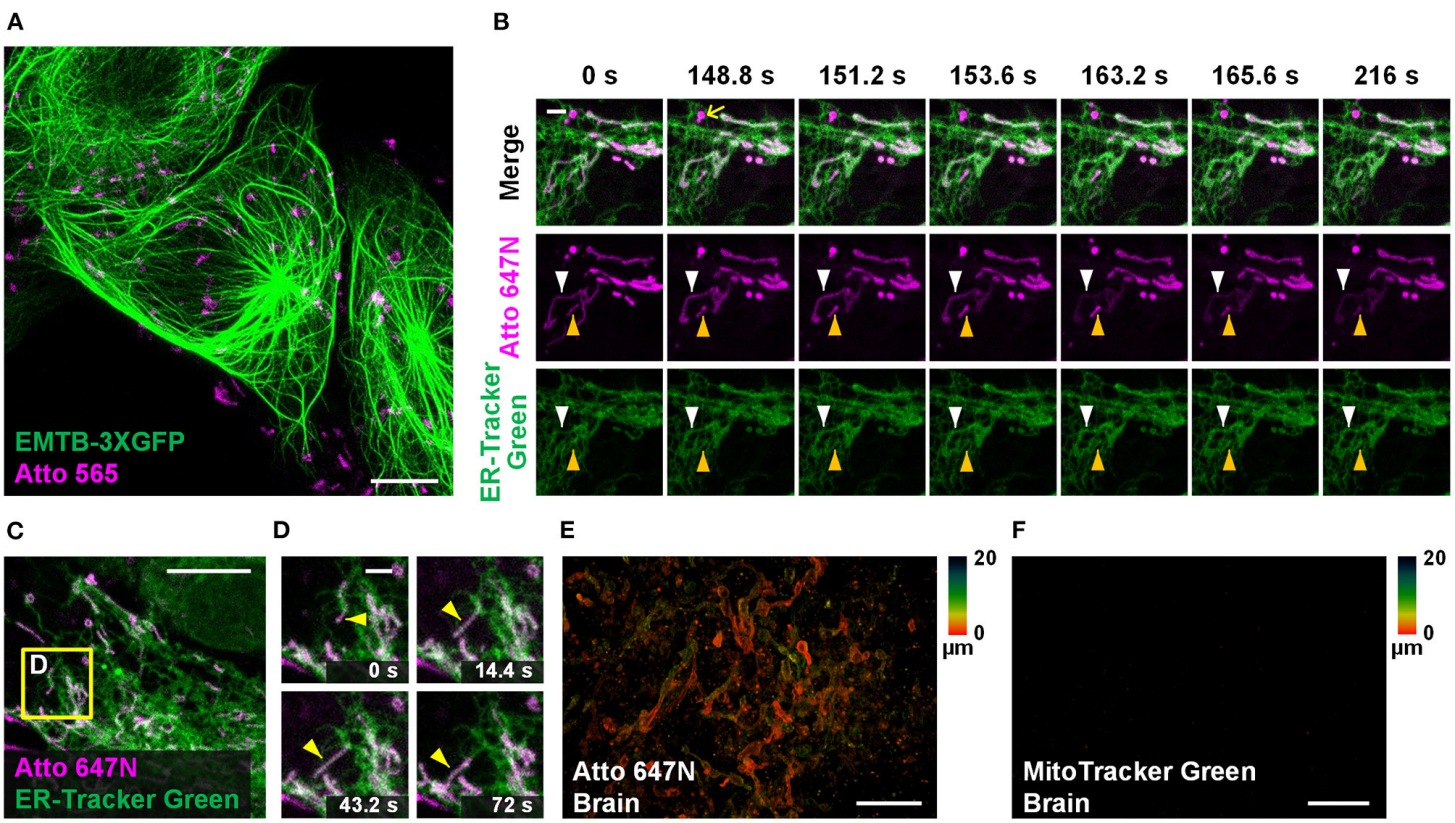
Other applications in living cells and tissues. **(A)** Dual-color confocal image (maximum intensity projection) of living U2OS cells labeled with EMTB-3XGFP (green) and Atto 565 (magenta; 3 μM). **(B,C)** Dual-color confocal image of living Astrocytes labeled with Atto 647N (magenta; 15 μM, middle row) and ER-Tracker Green (green; 2 μM, bottom row) for 30 min at 20°C. **(D)** Mitochondria move along ER tubules. No deconvolution or bleaching compensation procedure was applied during the figure rendering. For the time-lapse images, consecutive frames spaced at 2.4-s intervals were recorded; representative images of consecutive frames are displayed. Brain slices incubated with **(E)** Atto 647N and **(F)** MitoTracker Green. Color bars on the right of **(E)** and **(F)** indicate the imaging depth. Scale bars, **(A,C)** 10 μm, **(B,D)** 2 μm, and **(E,F)** 20 μm.

Dual-color staining combined with either fluorescent proteins (EMTB-3XGFP (Guo et al., 2018); Figure 5A) or other probes (ER-Tracker Green; Figures 5B–D and Supplementary Figure 14) were performed, suggesting the compatibility of our method with other labeling strategies. Long-term dual-color confocal imaging (20-min imaging duration at 2.4-s intervals) was performed in living Astrocytes, based on the excellent optical properties of Atto 647N (Figures 5B–D and Supplementary Figure 14). Interestingly, the results in astrocytes indicate that some of the ER substructures colocalized with mitochondria over time (the white and yellow arrowheads in Figure 5B; Supplementary Figure 14A). The possibility of crosstalk between channels was ruled out since there were some places with no overlap of the two channels (highlighted with the yellow arrows in Figure 5B). Moreover, hitchhiking interactions (Guo et al., 2018) between ER-mitochondria (Figures 5C,D), ER-vesicles (white arrowheads in Supplementary Figure 14C), and ER themselves (magenta arrowheads in Supplementary Figure 14C) were observed in our experiments.

### Labeling Living Tissues

Compared with typical mammalian cells cultured on coverslips, labeling living tissue samples usually harbor a more significant challenge. Tissues *in vitro* die soon after losing blood flow support, resulting in mitochondrial potential gradient loss. As a consequence, the cationic dyes cannot affine to mitochondria. To permit sufficient staining deep into the living tissue, the fluorescent probes should permeate through the tissues fast enough so that they can bind to mitochondria before the gradient is lost. Specifically, the brain slices were incubated with Atto 647N immediately after the mice were sacrificed. Our results suggest that 0.5-h incubation was sufficient for the dye to permeate for at least 20-μm thickness, indicating its excellent permeability through tissues (Figure 5E; Supplementary Figures 15A–C). In contrast, MitoTracker Green failed to stain the brain sample (Figure 5F), which suggests the abilities of Atto 647N to substitute MitoTracker probes in staining living tissues. Notably, our approach is highly versatile. Besides the brain slices, adipose tissues (Supplementary Figure 15D), blood cells (Supplementary Figure 15E), cardiac and skeletal muscle fibers (Supplementary Figures 15F,G), and embryo slices (Supplementary Figure 15H) were also successfully stained. Compared with the immunofluorescence techniques in previous works (Schneider Gasser et al., 2006; Chakrabarty et al., 2018), our method requires convenient and straightforward protocol steps, and direct incubation used here avoids the structural change caused by fixation. Therefore, our method is advantageous for many applications that expect instant actions, such as the rapid medical diagnoses of biospecimen *in vitro* and assessment for some mitochondrial diseases (Lee et al., 2019).

## Conclusion

In summary, the capability of many fluorescent dyes, which was ignored to some extent before, to stain various types of subcellular structures in living specimens with high brightness and photostability is confirmed. Potential targets include mitochondria, ER, endocytic vesicles, and the plasma membrane. The implementation requires only specific incubation conditions without any chemical modification or physical penetration, minimizing the damages and artifacts induced during the sample preparation. Moreover, Atto 647N exhibited extraordinary brightness and photostability in live-cell mitochondrial labeling, which can substitute SiR dye in long-term imaging or super-resolution microscopy.

Due to the limits from objective conditions, especially considering that the structures of many of the commercially available fluorescent dyes are a trade secret, not all of them are tested in this paper. It is worth noting that micromolar-level concentrations of some fluorophores may lead to a potential cytotoxicity to some sensitive cell lines. The organic solvent (e.g., DMSO) in the incubation solutions should also be controlled carefully to balance the dye dissolution and the toxicity to living cells when using new fluorophores. However, the hypothesis that was built in this work provides a guideline to elucidating these candidates and to broaden the applications of existing dyes. Moreover, the phenomena observed here show their great potential in answering a wide range of biological questions in living cells and tissues.

## Data Availability Statement

The raw data supporting the conclusions of this article will be made available by the authors, without undue reservation.

## Ethics Statement

The animal study was reviewed and approved by Zhejiang University.

## Author Contributions

XH, YH, CK, and XL conceived the project. Experiments were performed primarily by YH. ZZ and WL set up the system and contributed to the imaging. YH and YY prepared the samples. YH and XH drafted the manuscript. All authors contributed to the manuscript polish. All authors contributed to the article and approved the submitted version.

## Conflict of Interest

The authors declare that the research was conducted in the absence of any commercial or financial relationships that could be construed as a potential conflict of interest.
