# Supplementary material for: A Labeling Strategy for Living Specimens in Long-Term/Super-Resolution Fluorescence Imaging
Source: Front Chem. 2021 Jan 15;8:601436. doi: 10.3389/fchem.2020.601436 (PMC7843436; doi:10.3389/fchem.2020.601436)
Supplement: Supplementary file 1 [file Data_Sheet_1.docx]

Supplementary Material

[**Supplementary Figure 1**. Characterization of the Atto dyes in living cells. 2](#_Toc58247659)

[**Supplementary Figure 2**. Co-localization studies employing MitoTracker dyes as the standard mitochondrial markers. 3](#_Toc58247660)

[**Supplementary Figure 3**. Co-localization studies employing ZsGreen-Rab5 as the standard early endosomal marker. 4](#_Toc58247661)

[**Supplementary Figure 4**. Co-localization studies employing EGFP-Rab7A as the standard late endosomal marker. 5](#_Toc58247662)

[**Supplementary Figure 5**. Co-localization studies employing GFP-LAMP1 as the standard lysosomal marker. 6](#_Toc58247663)

[**Supplementary Figure 6**. Co-localization studies employing mRuby-Clathrin and pEGFP-Sec23A as the standard markers for endocytic-unassociated vesicular structures. 7](#_Toc58247664)

[**Supplementary Figure 7**. Endocytosis dependence of Atto 565 labeling. 8](#_Toc58247665)

[**Supplementary Figure 8**. pH sensitivity test of Atto 565. 9](#_Toc58247666)

[**Supplementary Figure 9**. Confocal images of living U2OS cells labeled with different dyes. 10](#_Toc58247667)

[**Supplementary Figure 10**. Co-localization studies for Cy5 and BODIPY 650/665. 11](#_Toc58247668)

[**Supplementary Figure 11**. Confocal images of different cell lines labeled with the Atto dyes. 12](#_Toc58247669)

[**Supplementary Figure 12**. Cell viabilities of different cell lines stained with the Atto dyes. 13](#_Toc58247670)

[**Supplementary Figure 13**. The first and last frames from 20-min confocal imaging of different probes. 16](#_Toc58247671)

[**Supplementary Figure 14**. Dual-color confocal images of living Astrocytes. 17](#_Toc58247672)

[**Supplementary Figure 15**. Applications in different living brain slices. 18](#_Toc58247673)

[**Supplementary Table 1.** Recommended conditions for live-cell labeling using the live-cell incompatible dyes. 19](#_Toc58247674)

[**Supplementary Table 2**. Experimental conditions for long-term confocal imaging. 19](#_Toc58247675)

[**Supplementary Table 3**. Comparison of the optical properties of frequently-used red-absorbing fluorescent dyes. 20](#_Toc58247676)


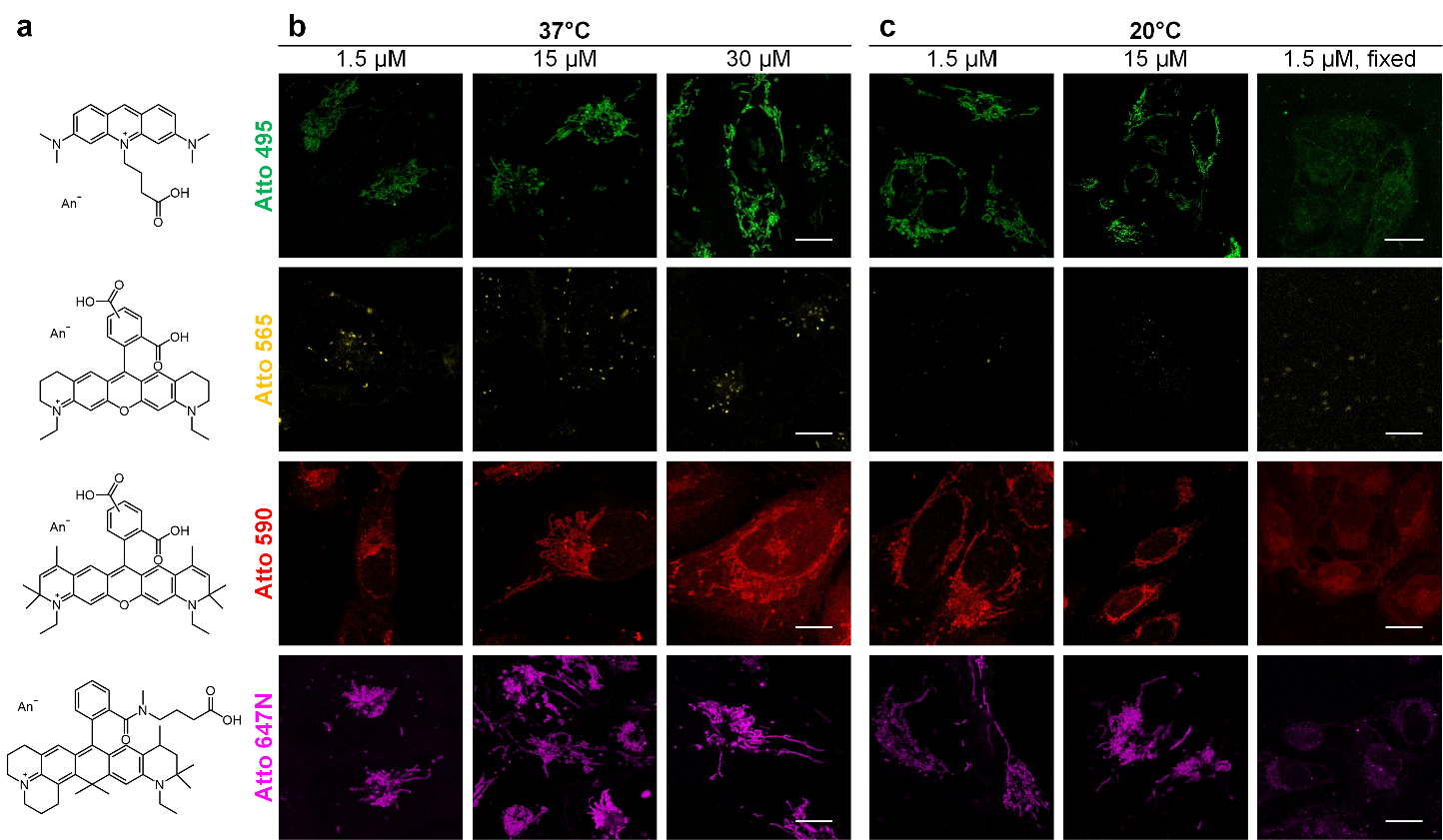


**Supplementary Figure 1**. Characterization of the Atto dyes in living cells.

(**a**) Chemical structures of Atto 495, Atto 565, Atto 590, Atto 647N. Confocal images of living U2OS cells incubated with the Atto dyes at either (**b**) 37°C or (**c**) 20°C for 30 min. The fixation was accomplished by incubation with 4% paraformaldehyde for 10 min. Scale bars: 10 μm.


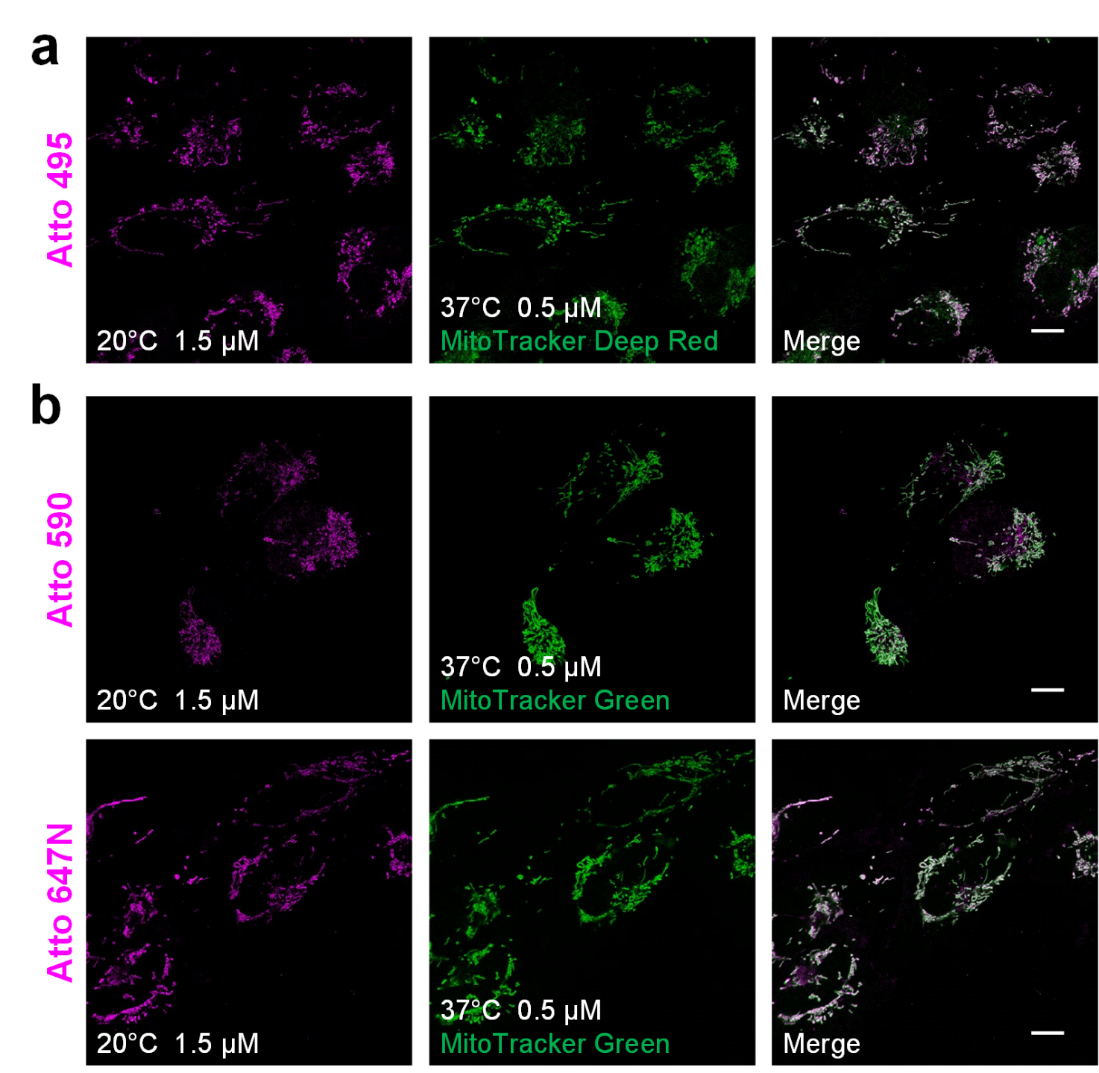


**Supplementary Figure 2**. Co-localization studies employing MitoTracker dyes as the standard mitochondrial markers.

(**a**) Living U2OS cells were incubated with Atto 495 (magenta, 1.5 μM) for 30 min at 20°C and then with MitoTracker Deep Red (green, 0.5 μM) for 30 min at 37°C before imaging. (**b**) Living U2OS cells were incubated with Atto 590 or Atto 647N (magenta, 1.5 μM) for 30 min at 20°C and then with MitoTracker Green (green, 0.5 μM) for 30 min at 37°C before imaging. Scale bars: 10 μm.


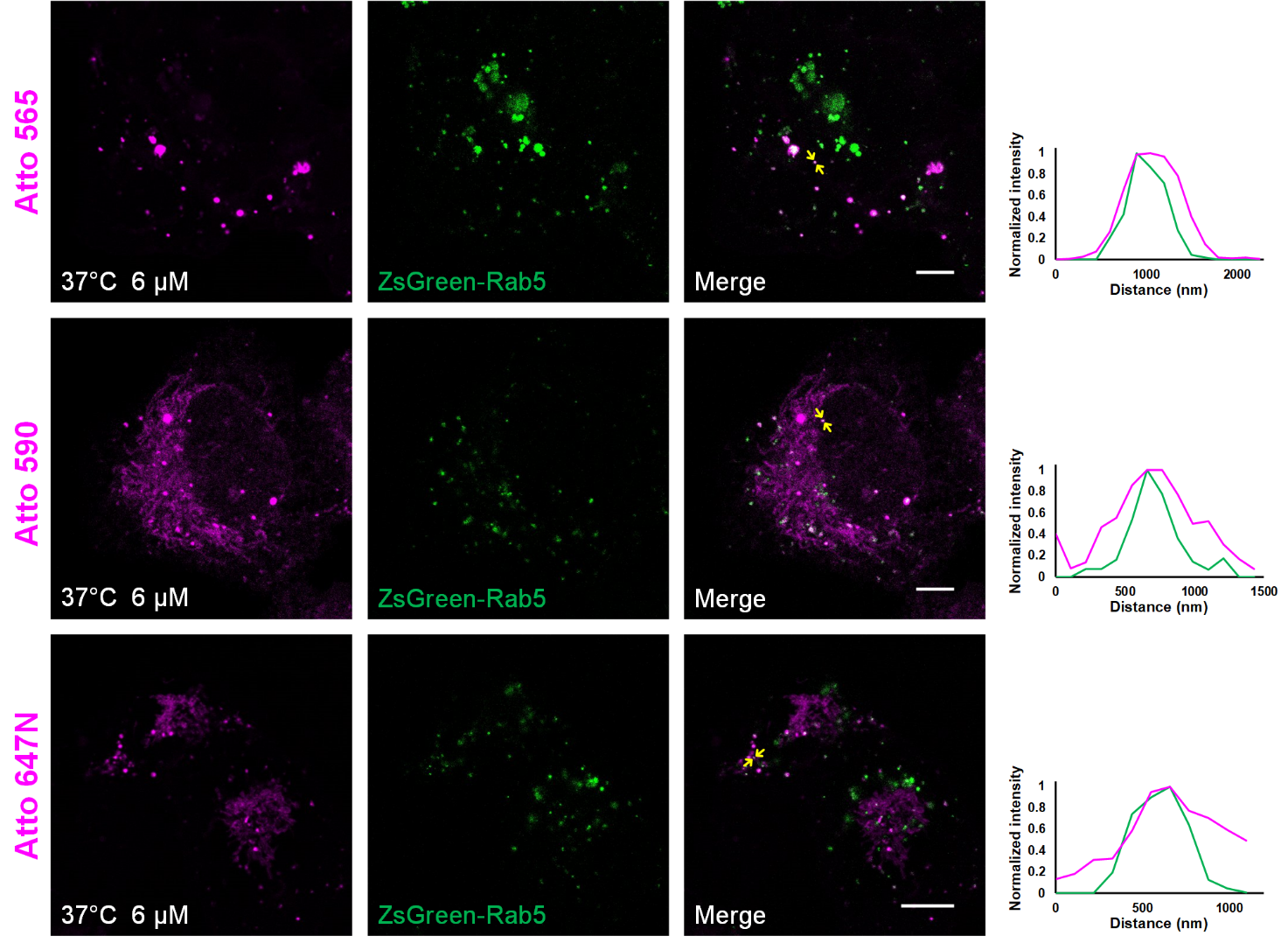


**Supplementary Figure 3**. Co-localization studies employing ZsGreen-Rab5 as the standard early endosomal marker.

Living U2OS cells transiently transfected by ZsGreen-Rab5 (green, the second column from left) were stained with Atto 565, Atto 590, or Atto 647N (magenta, 6 µM, the first column) for 30 min at 37°C and imaged by confocal microscope. On the right, the intensity profiles at the position denoted by the yellow arrows in the merged image (the third column from left) are shown, indicating the co-localization of the Atto dyes and the early endosomes. Scale bars: 10 μm.


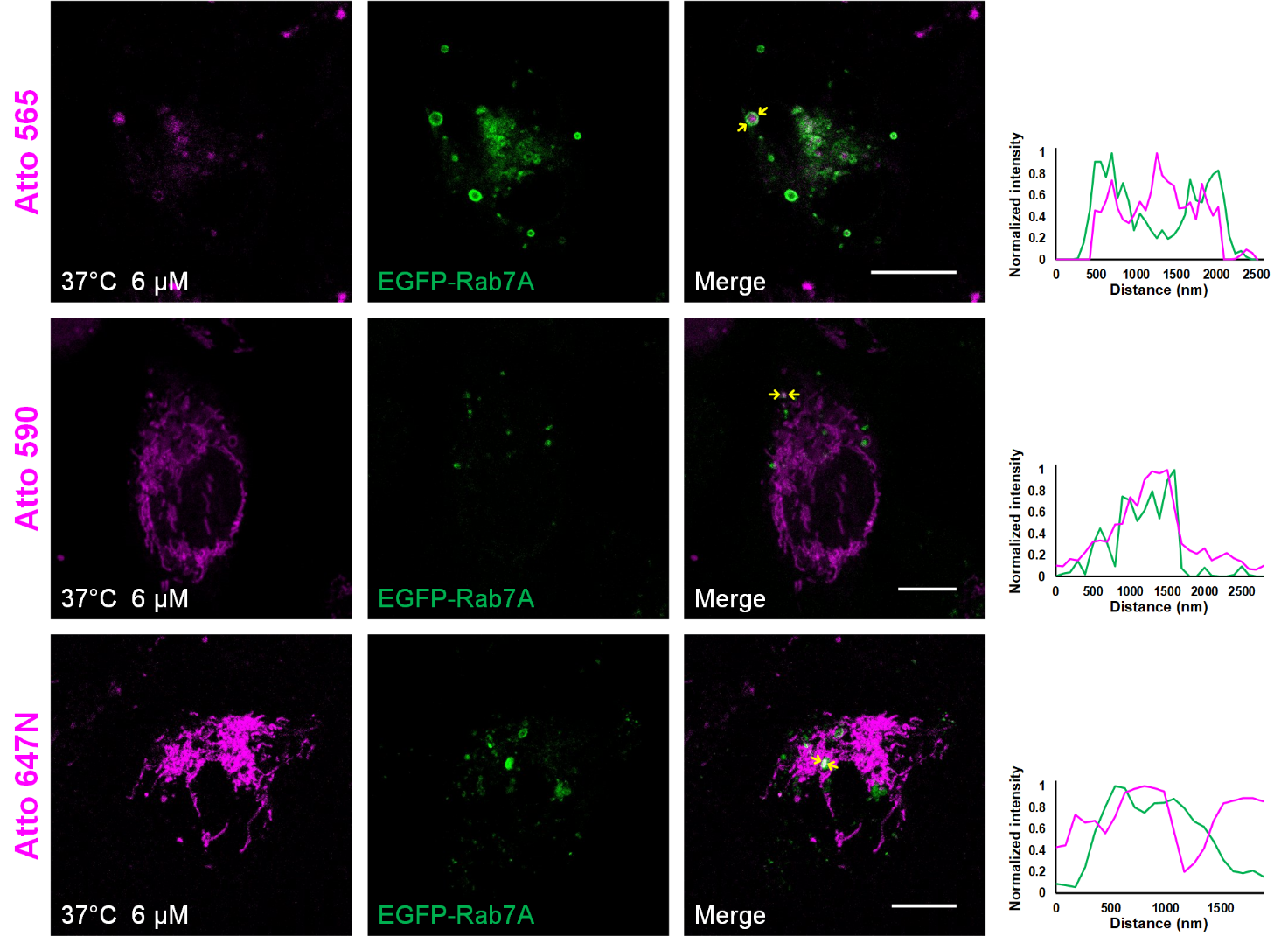


**Supplementary Figure 4**. Co-localization studies employing EGFP-Rab7A as the standard late endosomal marker.

Living U2OS cells transiently transfected by EGFP-Rab7A (green, the second column from left) were stained with Atto 565, Atto 590, or Atto 647N (magenta, 6 µM, the first column) for 30 min at 37°C and imaged by confocal microscope. On the right, the intensity profiles at the position denoted by the yellow arrows in the merged image (the third column) are shown, indicating the co-localization of the Atto dyes and the late endosomes. Scale bars: 10 μm.


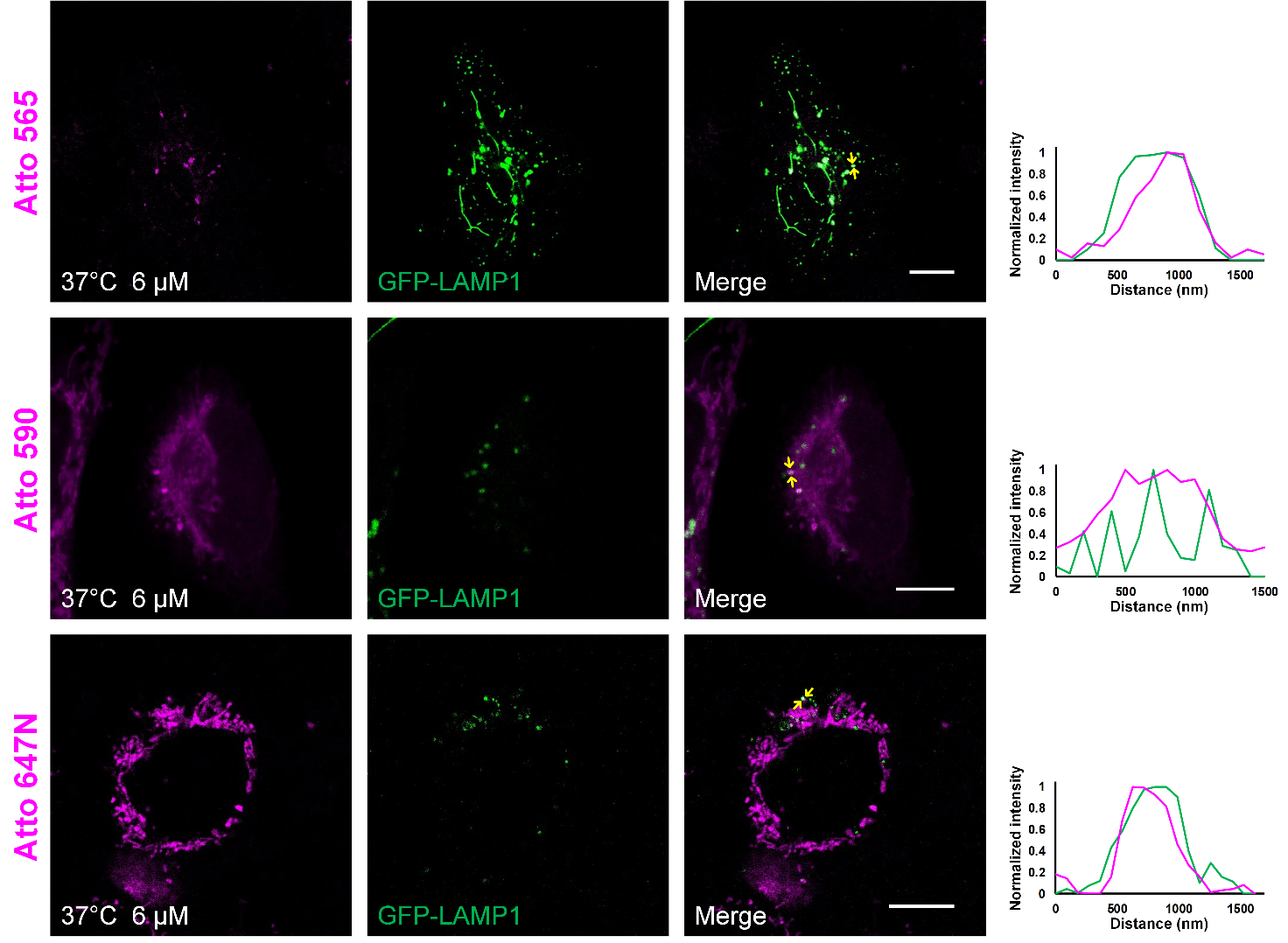


**Supplementary Figure 5**. Co-localization studies employing GFP-LAMP1 as the standard lysosomal marker.

Living U2OS cells transiently transfected by GFP-LAMP1 (green, the second column from left) were stained with Atto 565, Atto 590, or Atto 647N (magenta, 6 µM, the first column) for 30 min at 37°C and imaged by confocal microscope. On the right, the intensity profiles at the position denoted by the yellow arrows in the merged image (the third column) are shown, indicating the co-localization of the Atto dyes and the lysosomes. Scale bars: 10 μm.


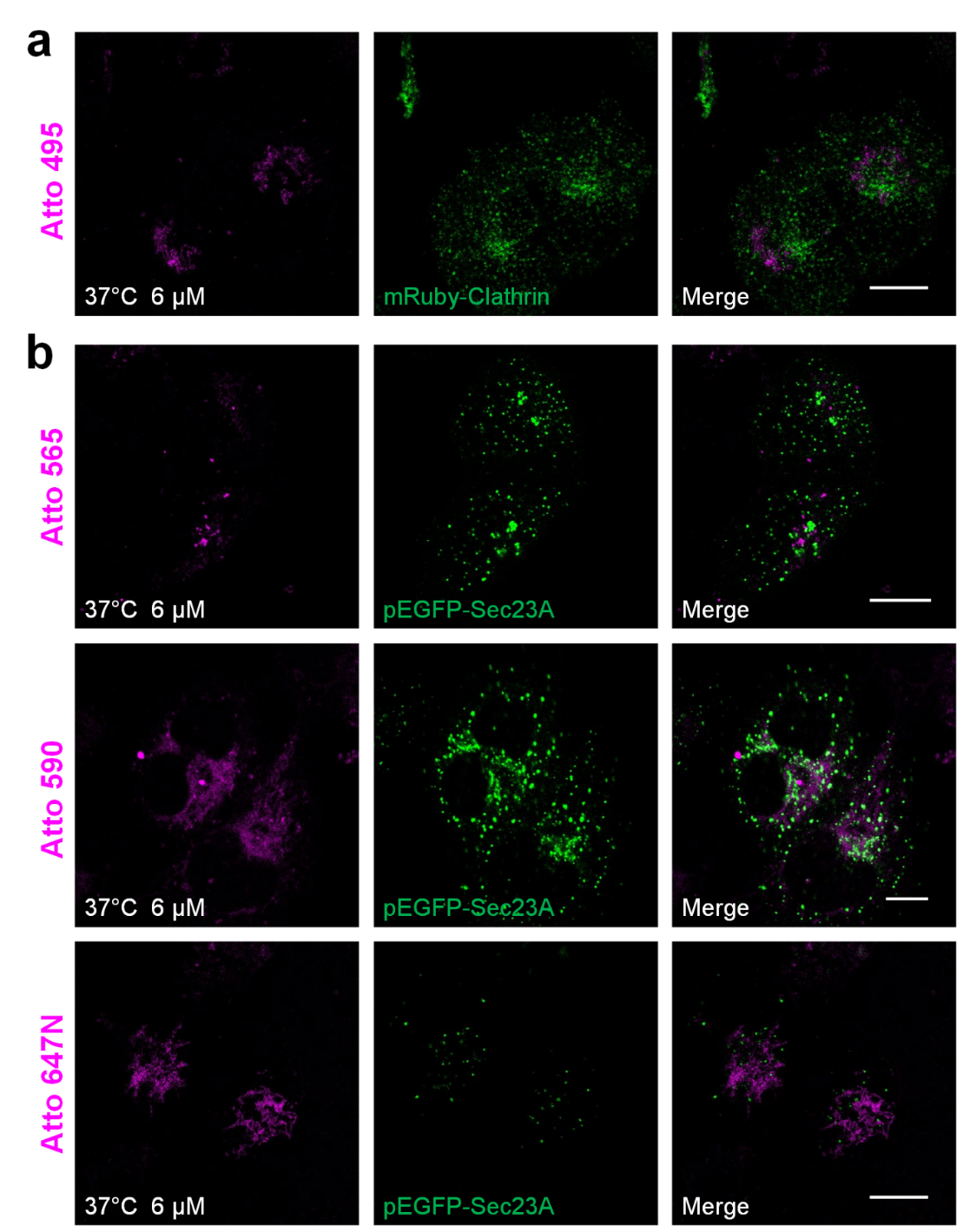


**Supplementary Figure 6**. Co-localization studies employing mRuby-Clathrin and pEGFP-Sec23A as the standard markers for endocytic-unassociated vesicular structures.

(**a**) Living U2OS cells transiently transfected by mRuby-Clathrin (green, the middle sub-figure) were stained with Atto 495 (magenta, 6 µM, the left sub-figure) for 30 min at 37°C and imaged by confocal microscope. Two images were then merged and shown as the sub-figure on the right. Pearson correlation coefficient: 0.15. Similarly, in (**b**), living U2OS cells transiently transfected by pEGFP-Sec23A (green, the middle column) were stained with Atto 565, Atto 590, or Atto 647N (magenta, 6 µM, the left column) for 30 min at 37°C and imaged by confocal microscope. Pearson correlation coefficient: 0.18, 0.0.41, and 0.05 for Atto 565, Atto 590, and Atto 647N, respectively. The merged images were shown on the right in each row. Scale bars: 10 μm.


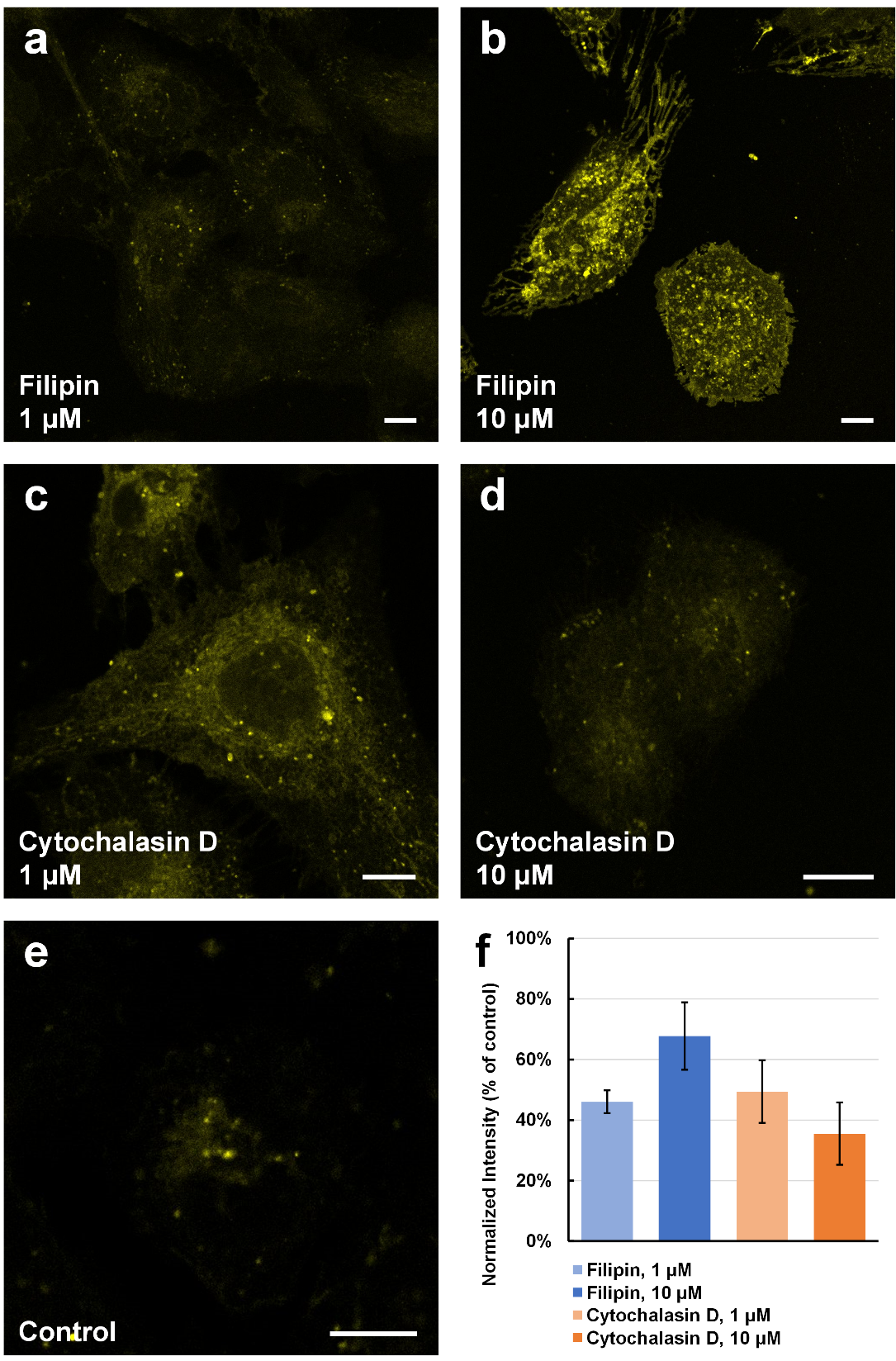


**Supplementary Figure 7**. Endocytosis dependence of Atto 565 labeling.

Living U2OS cells were treated with either (**a, b**) Filipin or (**c, d**) Cytochalasin D for 30 min and then labeled with Atto 565 at 37°C. (**e**) Control group without drug treatment before Atto 565 labeling. (**f**) The mean fluorescence intensity of Atto 565 labeling after the drug treatment. Error bars represent the standard deviations of at least triplicate experiments. Scale bars: 10 μm.


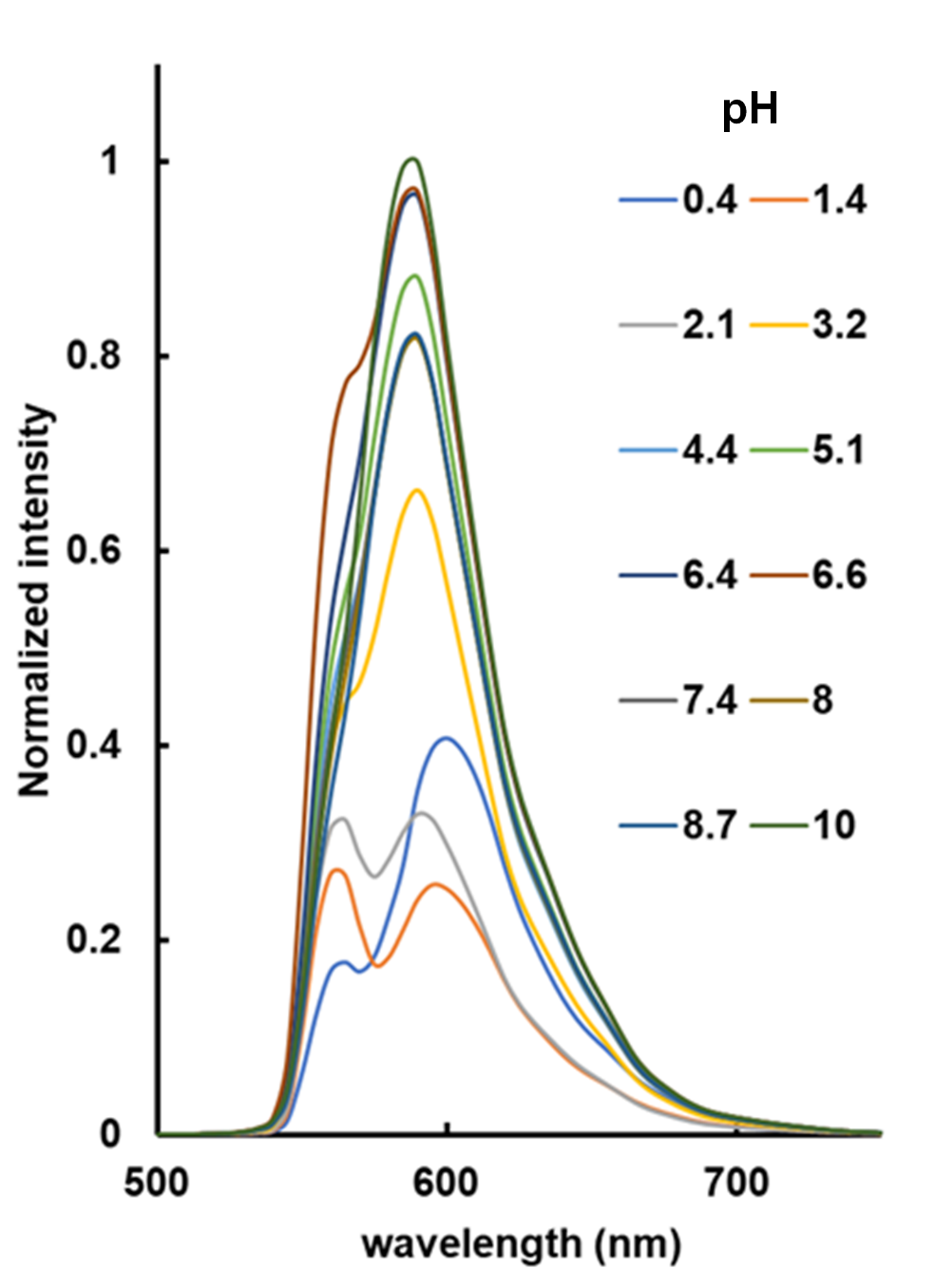


**Supplementary Figure 8**. pH sensitivity test of Atto 565.

Fluorescence excitation spectra of Atto 565 (λ_ex_ = 563 nm) at different pH. The curves were normalized by dividing with the maximum of all the fluorescence intensities acquired.


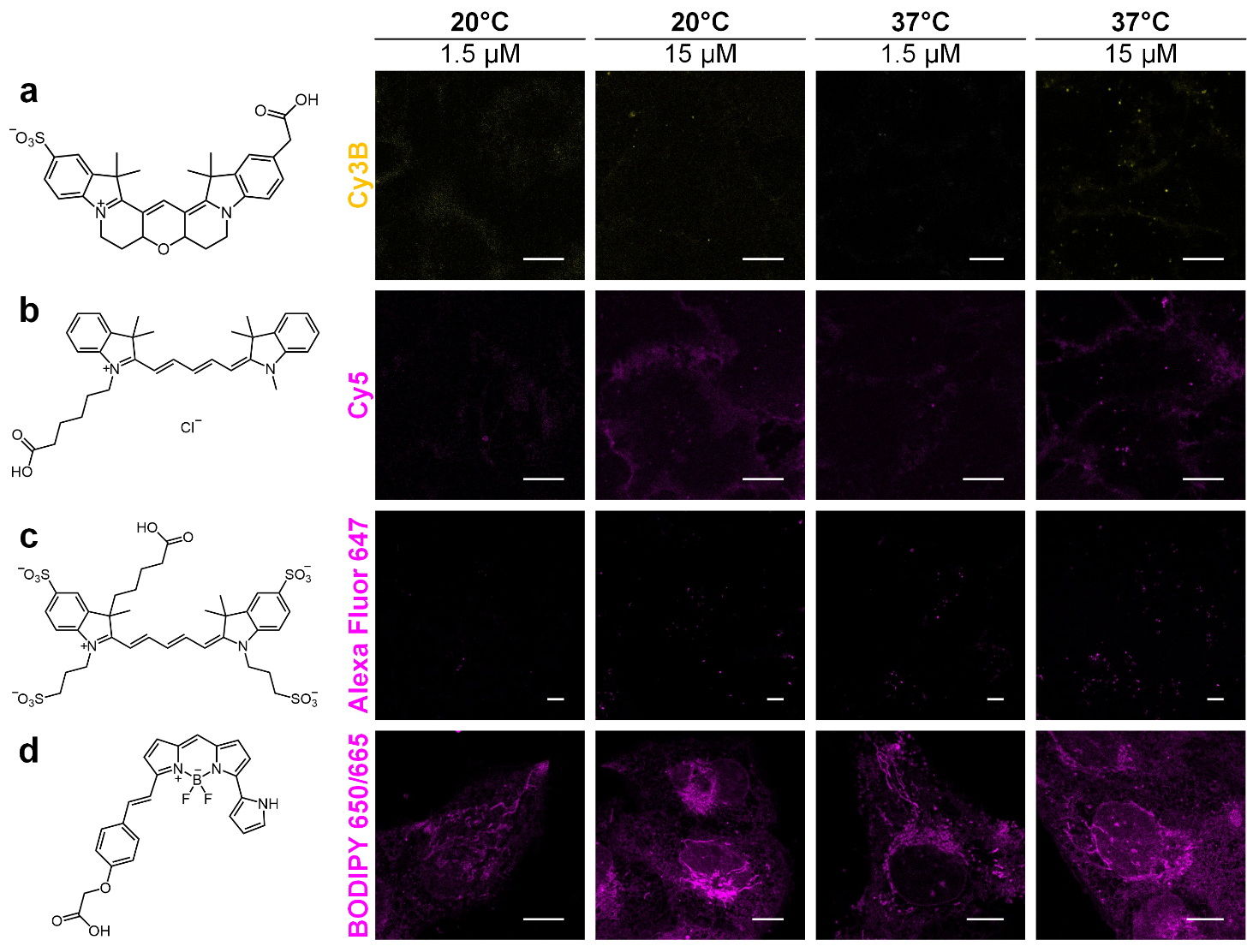


**Supplementary Figure 9**. Confocal images of living U2OS cells labeled with different dyes.

Cells were incubated with (**a**) Cy3B, (**b**) Cy5, (**c**) Alexa Fluor 647, and (**d**) BODIPY 650/665 at different concentrations (1.5 μM or 15 μM) and temperatures (20°C or 37°C). Scale bars: 10 μm.


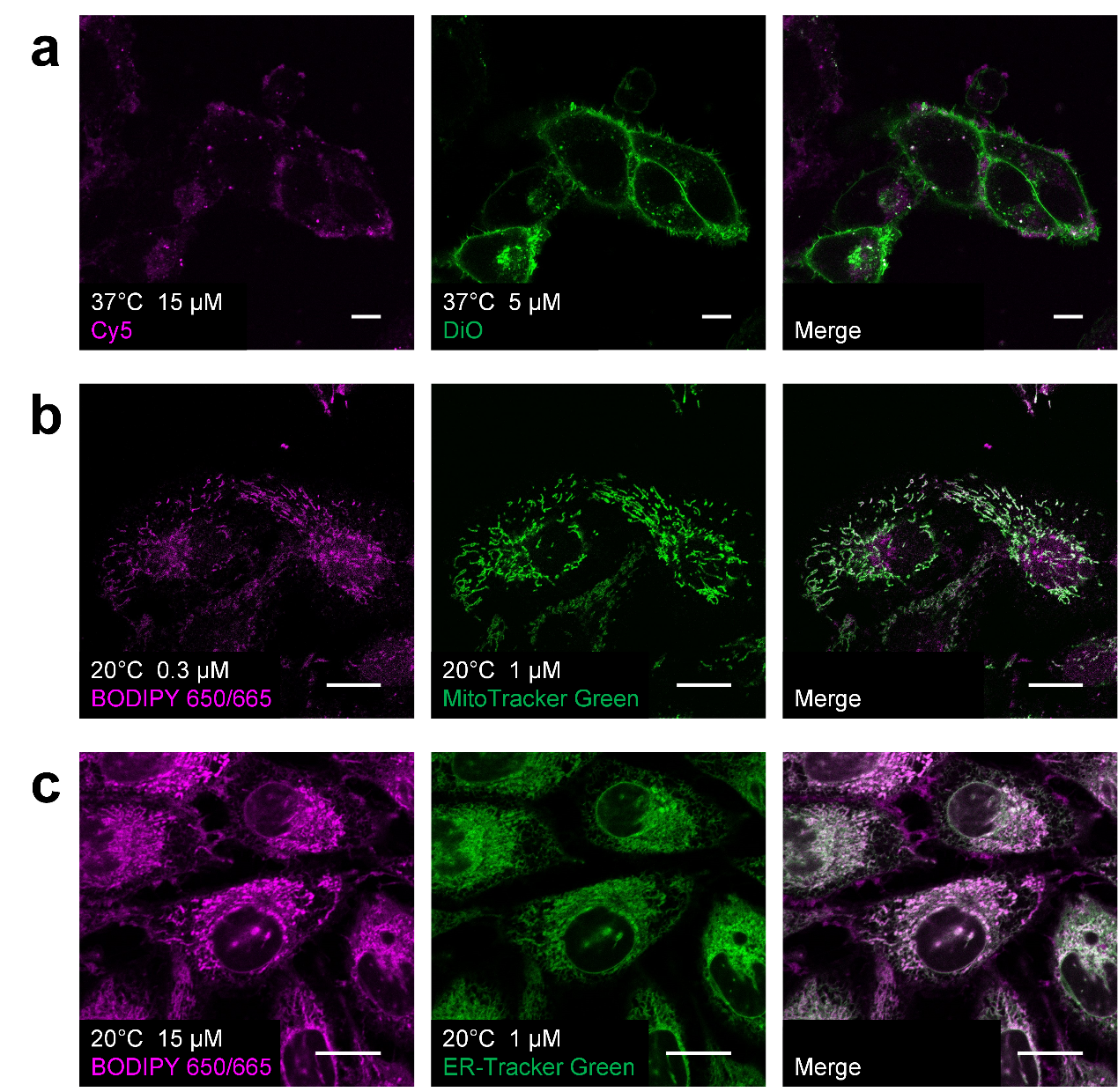


**Supplementary Figure 10**. Co-localization studies for Cy5 and BODIPY 650/665.

(**a**) Living U2OS cells were stained with Cy5 (magenta, 15 µM) and DiO (green, 5 µM) for 30 min at 37°C and imaged by confocal microscope. Two images were then merged and shown as the sub-figure on the right. Pearson correlation coefficient: 0.41. (**b**) Living U2OS cells were stained with BODIPY 650/665 (magenta, 0.3 µM) and MitoTracker Green (green, 1 µM) for 30 min at 20°C and imaged by confocal microscope. Two images were then merged and shown as the sub-figure on the right. Pearson correlation coefficient: 0.52. (**c**) Living U2OS cells were stained with BODIPY 650/665 (magenta, 15 µM) and ER-Tracker Green (green, 1 µM) for 30 min at 20°C and imaged by confocal microscope. Two images were then merged and shown as the sub-figure on the right. Pearson correlation coefficient: 0.82. Scale bars: 10 μm.


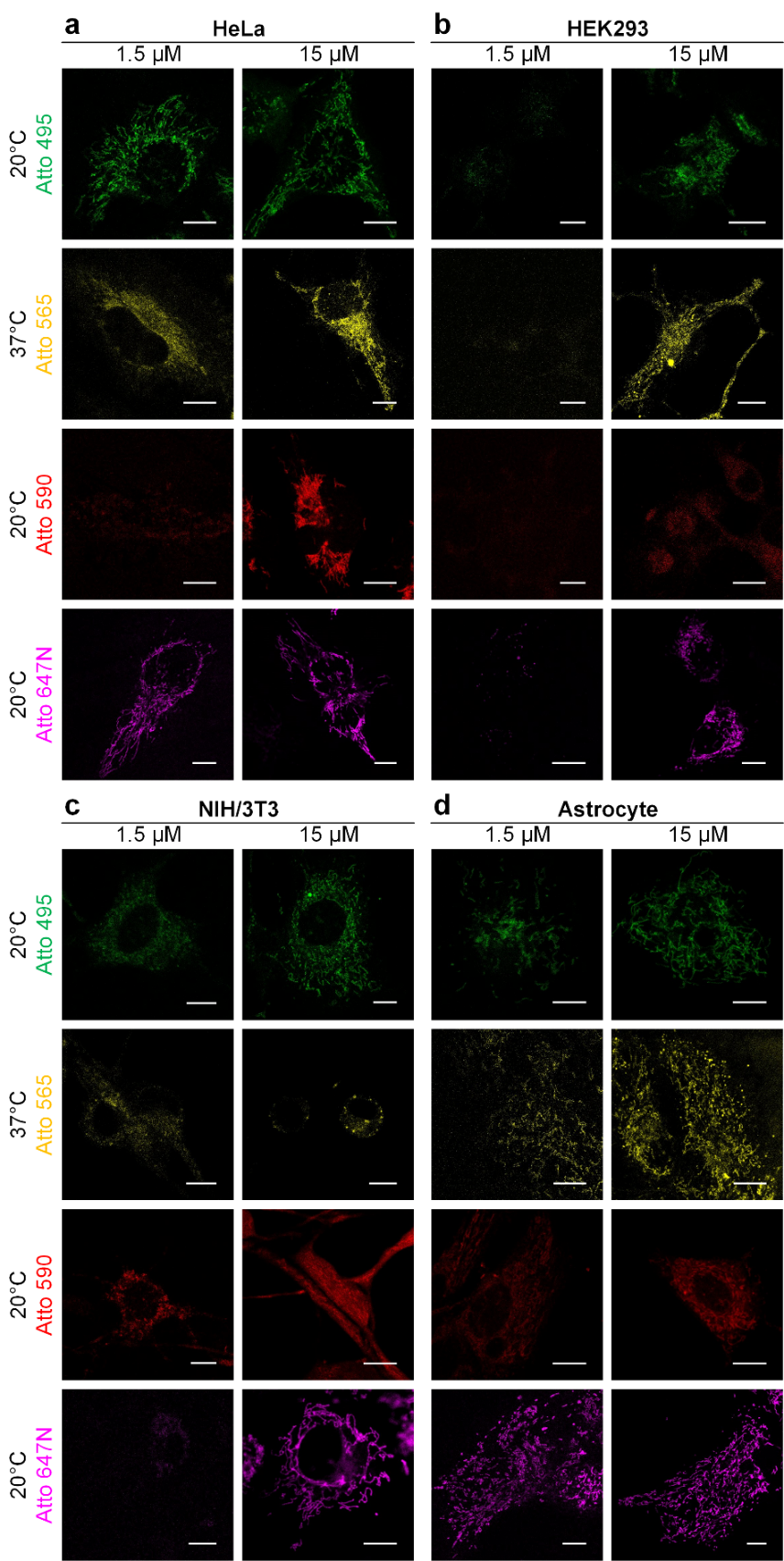


**Supplementary Figure 11**. Confocal images of different cell lines labeled with the Atto dyes.

Living (**a**) HeLa, (**b**) HEK293, (**c**) NIH/3T3, or (**d**) Astrocyte cells were incubated with Atto 495 (at 20°C), Atto 565 (at 37°C), Atto 590 (at 20°C), and Atto 647N (at 20°C) for 30 min. Scale bars: 10 μm.


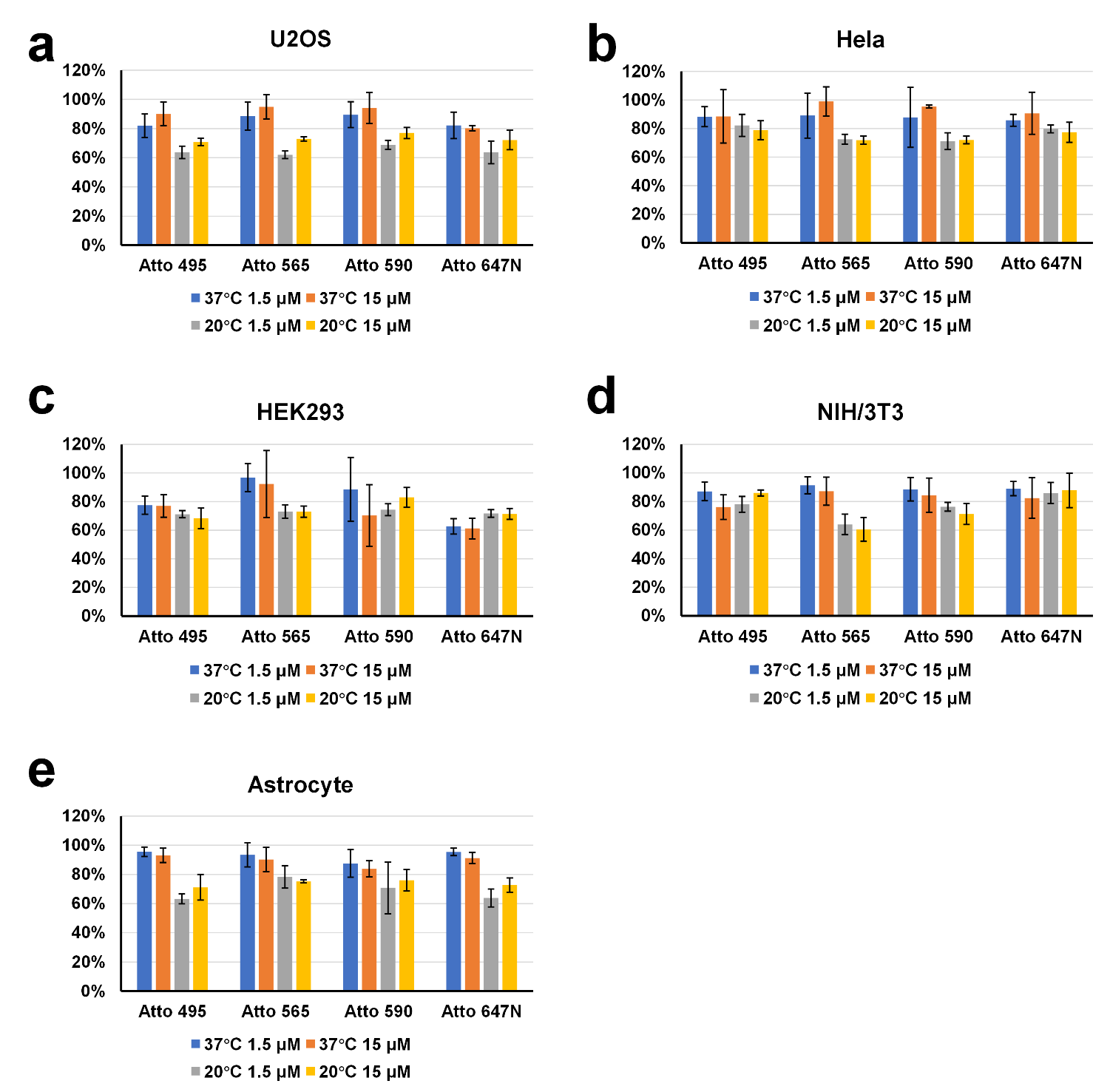


**Supplementary Figure 12**. Cell viabilities of different cell lines stained with the Atto dyes.

Different living cells, (**a**) U2OS, (**b**) HeLa, (**c**) HEK293, (**d**) NIH/3T3, or (**e**) Astrocyte, were incubated with the Atto dyes at 20°C or 37°C for 30 min. Error bars represent the standard deviations of triplicate experiments.


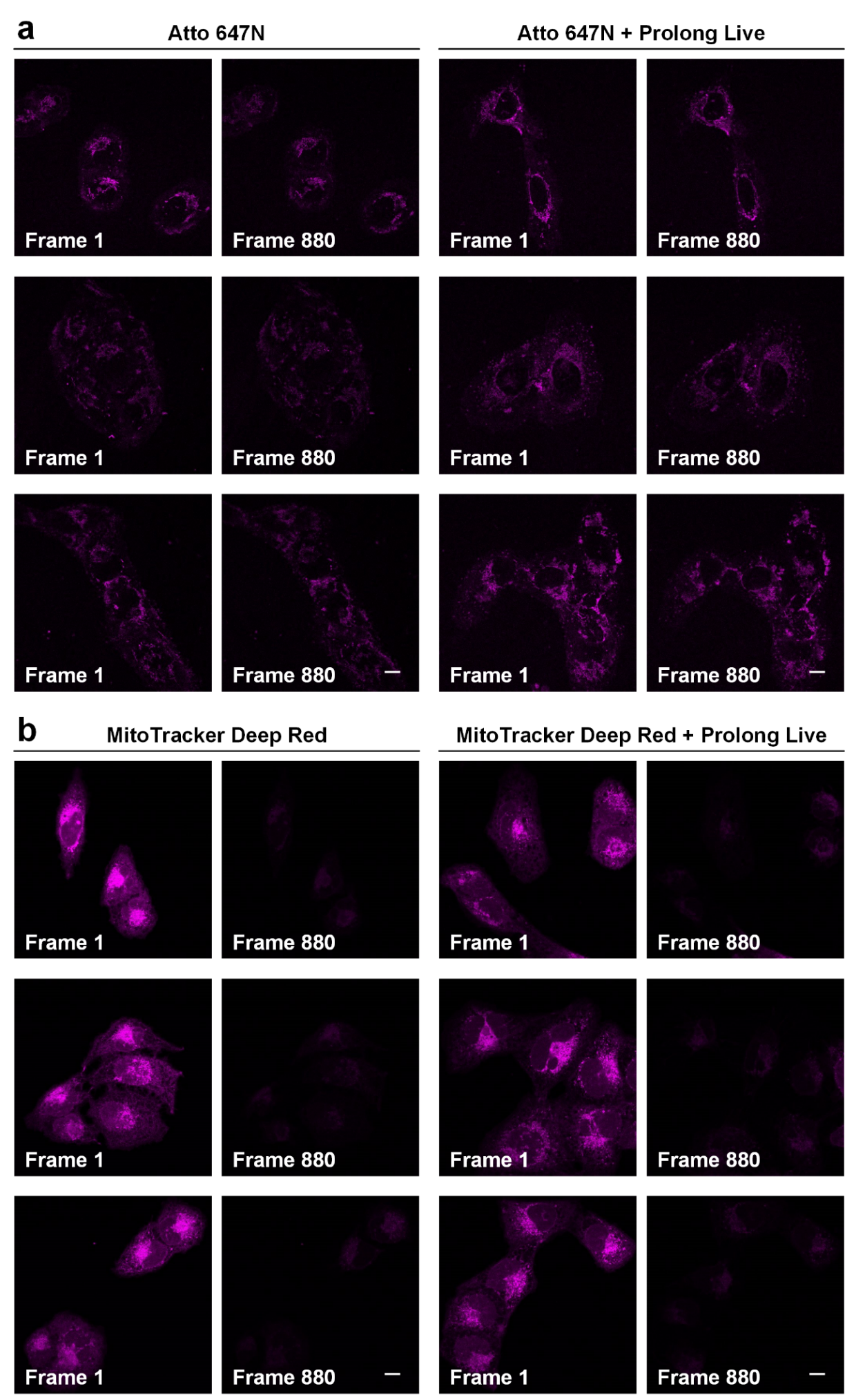


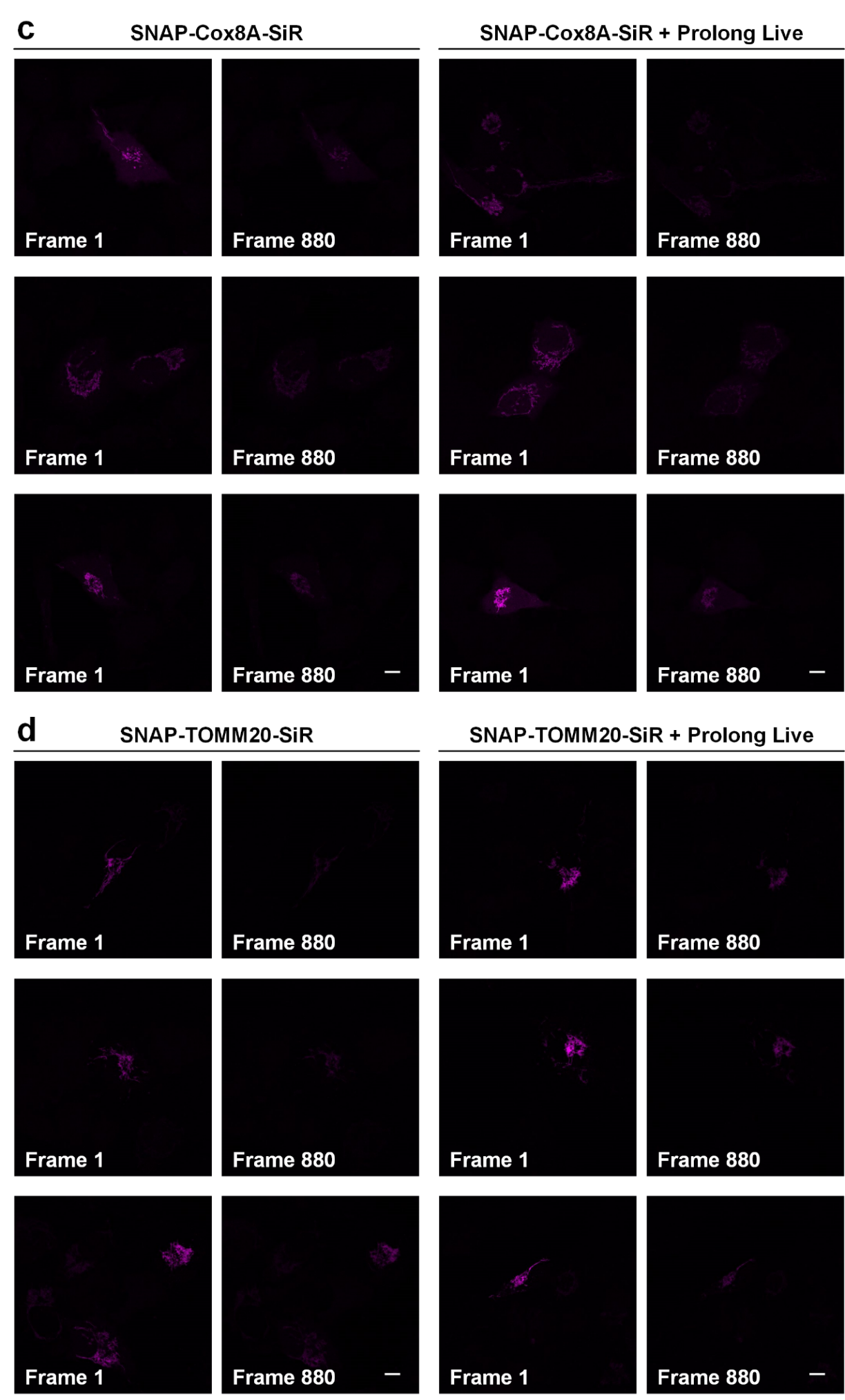


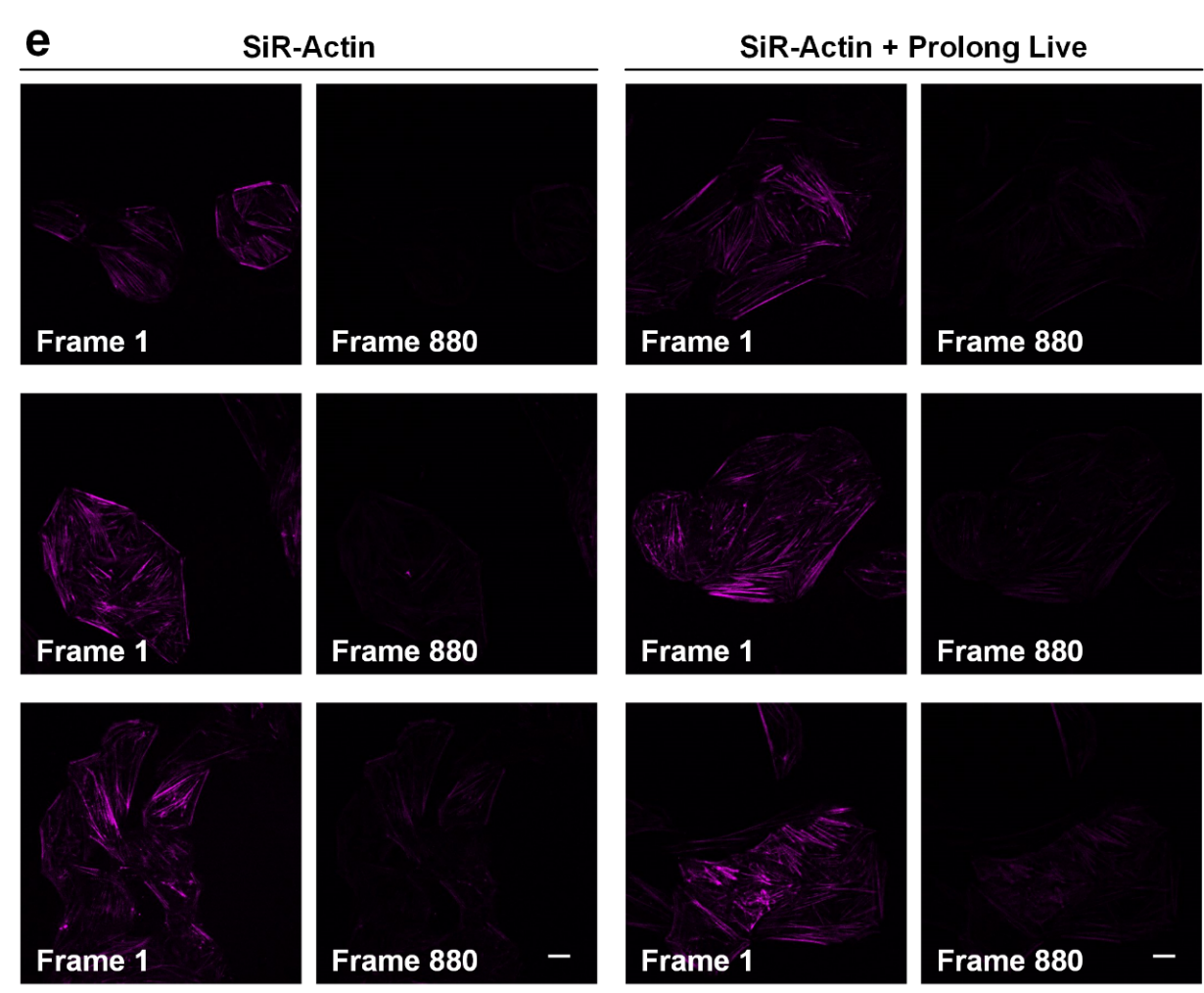


**Supplementary Figure 13**. The first and last frames from 20-min confocal imaging of different probes.

Living U2OS cells were labeled with (**a**) Atto 647N, (**b**) MitoTracker Deep Red, (**c**) SNAP-Cox8A-SiR, (**d**) SNAP-TOMM20-SiR, or (**e**) SiR-Actin. Scale bars: 10 μm.


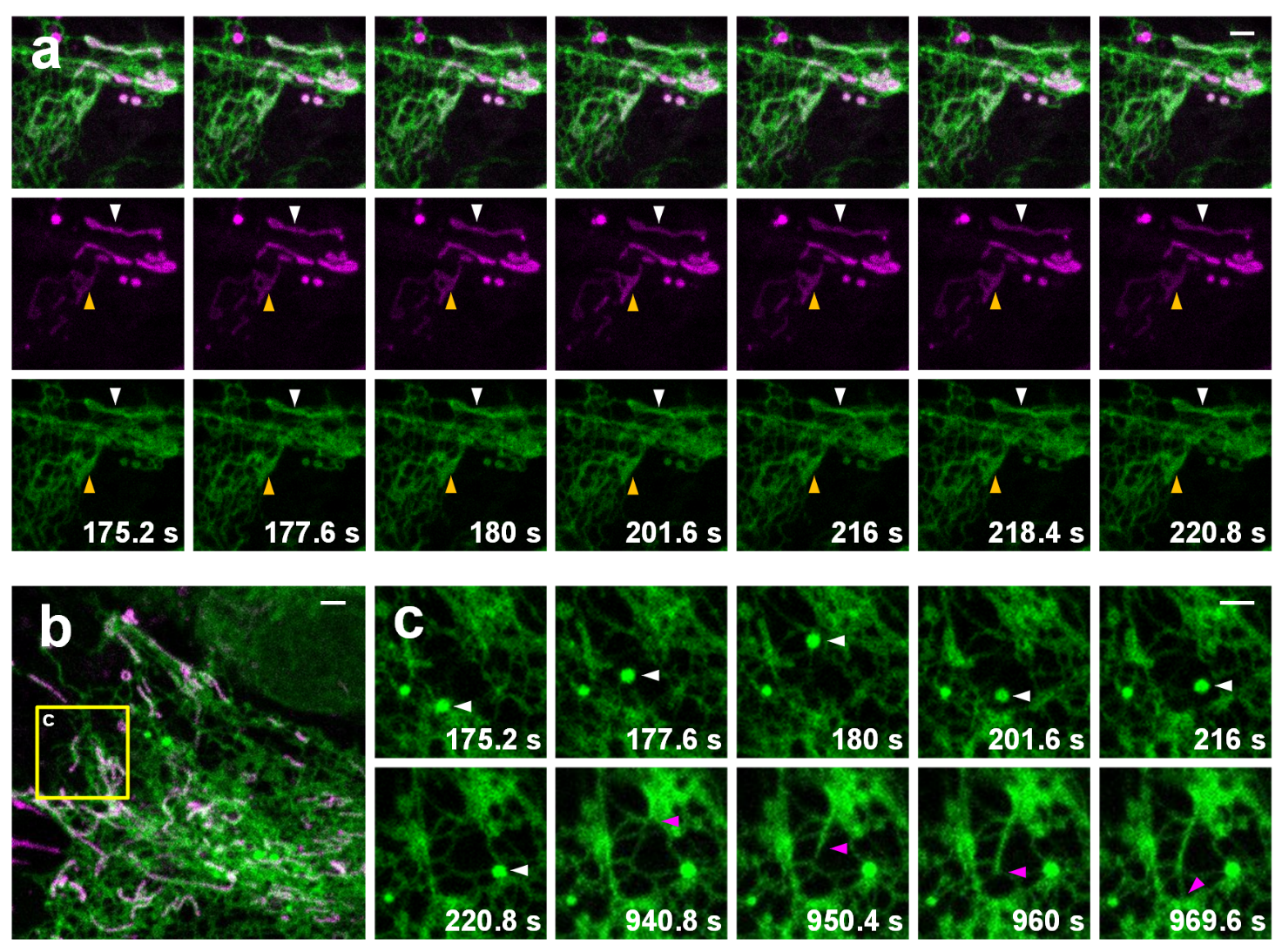


**Supplementary Figure 14**. Dual-color confocal images of living Astrocytes.

(**a, b**) The cells were labeled with ER-Tracker Green (green; 2 μM) and Atto 647N (magenta; 15 μM) for 30 min at 20°C. (**c**) Vesicles or ER tubules move along ER tubules. For the time-lapse images, consecutive frames spaced at 2.4-s intervals were obtained; representative images of consecutive frames are displayed. Scale bars: 2 μm.


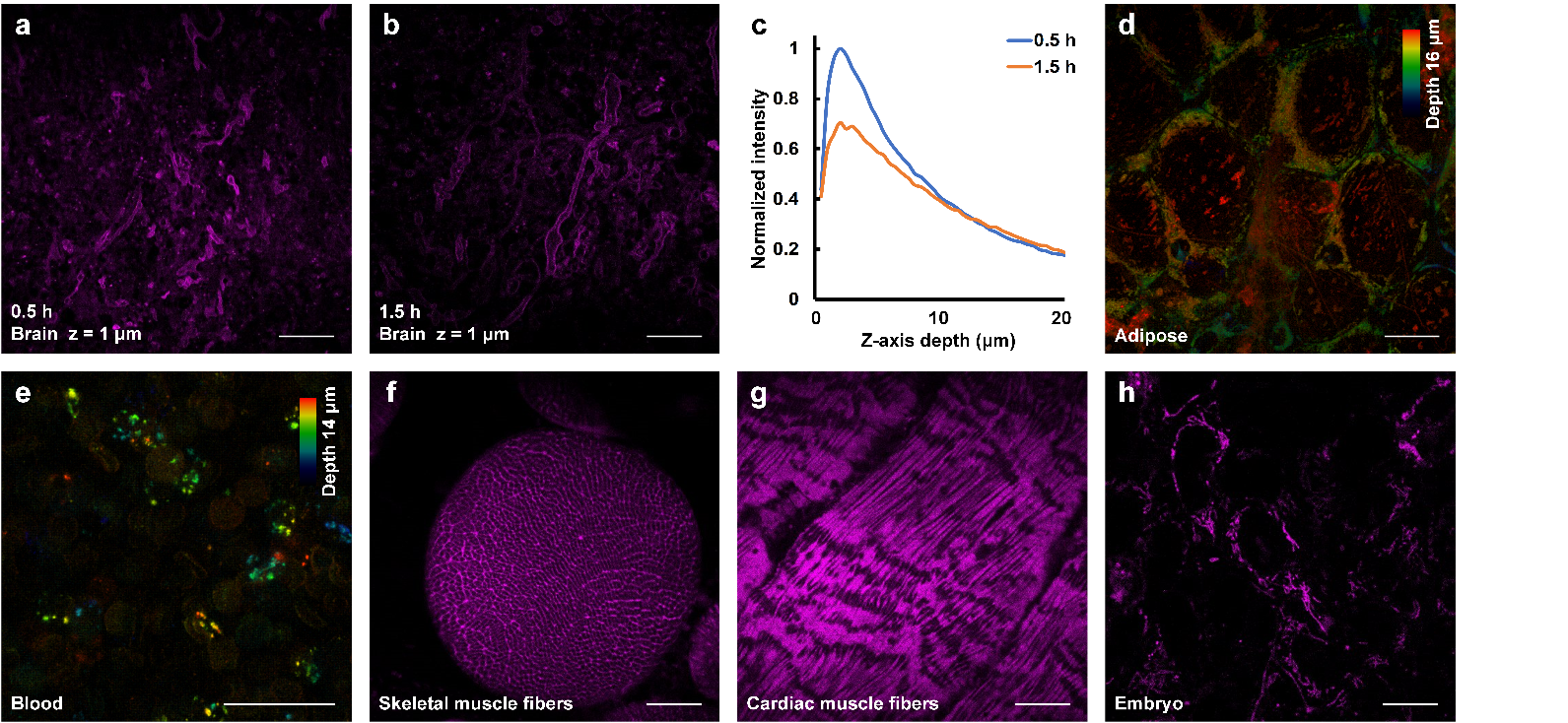


**Supplementary Figure 15**. Applications in different living brain slices.

The 3^rd^ frames (*z* = 1 μm) of the brain slices incubated with Atto 647N for (**a**) 0.5 h or (**b**) 1.5 h. (**c**) Intensity profiles along the *z*-axis of the brain slice in **a** and **b**. (**d**) adipose tissue, (**e**) blood cells, (**f**) Skeletal muscles, (**e**) cardiac muscles, and (**f**) embryo slices labeled with Atto 647N. Scale bars: (**a,** **b**, **d**, **f-h**) 20 μm; (**e**) 10 μm.

**Supplementary Table 1.** Recommended conditions for live-cell labeling using the live-cell incompatible dyes.

| ***Subcellular structures*** | ***Electric charges*** | ***Additional properties*** | ***Incubation concentration*** | ***Incubation temperature*** | ***Recommended dyes*** |
| --- | --- | --- | --- | --- | --- |
| ***Mitochondria*** | Cationic dyes | Moderate cell permeability | 1.5-15 μM | 20°C | Atto 647N |
| ***ER*** | zwitterionic BODIPY dyes | Higher cell permeabilities or dosages | 15 μM | 20°C | BODIPY 650/665 |
| ***Endocytic vesicles*** | - | Particularly low cell-permeability | 1.5-15 μM | 37°C | AF 647, Cy3B |

**Supplementary Table 2**. Experimental conditions for long-term confocal imaging.

| ***Label*** | ***Dosage (µM)*** | ***Incubation temperature (°C)*** | ***Incubation time (min)*** | ***Excitation λ (nm)*** | ***Illumination intensity (kW/cm^2^)*** |
| --- | --- | --- | --- | --- | --- |
| ***Atto 647N*** | 1.5 | 20 | 30 | 640 | 1.25 |
| ***MitoTracker Deep Red*** | 0.2 | 37 |  |  |  |
| ***SNAP-Cell 647-SiR*** | 3 | 37 |  |  |  |
| ***Actin-SiR*** | 1 | 37 |  |  |  |
| ***Dimension*** | ***Pixel size (µm)*** | ***Exposure time per raw image (ms)*** | ***Pinhole size (µm)*** | ***Time points*** | ***Cycle time (Acquisition + resting time) (s)*** |
| 512 × 512 | 0.25 | 293.92 | 30.00 | 880 | 1.36 |

**Supplementary Table 3**. Comparison of the optical properties of frequently-used red-absorbing fluorescent dyes.

| **Dye** | **Excitation Maximum (nm)^a^** | **Emission Maximum (nm)^a^** | **Extinction (m^-1^ cm^-1^)^b^** | **Quantum Yield^c^** | **Brightness^d^** |
| --- | --- | --- | --- | --- | --- |
| **Atto 647N** | 644 | 669 | 150,000 | 0.65 | 97,500 |
| **MitoTracker Deep Red FM** | 644 | 665 | - | - | - |
| **SiR** | 645 | 661 | 100,000 | 0.39 | 39,000 |
| **BODIPY 650/665** | 646 | 660 | 102,000 | 0.46 | 46,920 |
| **Cy5** | 649 | 670 | 250,000 | 0.28 | 70,000 |
| **STAR 635P** | 634 | 654 | 120,000 | 0.90 | 108,000 |
| **STAR 635** | 635 | 659 | 120,000 | 0.25 | 30,000 |
| **Alexa Fluor 647** | 650 | 665 | 239,000 | 0.33 | 78,870 |
| **Atto 647** | 645 | 669 | 120,000 | 0.20 | 24,000 |

^a^ Excitation and emission peak wavelengths of dye spectra.

^b^ Extinction coefficients reported by the dye manufacturers.

^c^ Quantum yields from either the dye manufacturer or from the McNamara fluorophore data tables. -, values not available from the dye manufacturer or McNamara data tables.

^d^ Brightness = Extinction × Quantum Yield.
